# Seeking a unified framework for cerebellar function and dysfunction: from circuit operations to cognition

**DOI:** 10.3389/fncir.2012.00116

**Published:** 2013-01-10

**Authors:** Egidio D'Angelo, Stefano Casali

**Affiliations:** ^1^Department of Brain and Behavioral SciencesPavia, Italy; ^2^IRCCS C. Mondino, Brain Connectivity CenterPavia, Italy

**Keywords:** cerebellum, cognition, motor control, timing, prediction, autism, schizophrenia, dyslexia

## Abstract

Following the fundamental recognition of its involvement in sensory-motor coordination and learning, the cerebellum is now also believed to take part in the processing of cognition and emotion. This hypothesis is recurrent in numerous papers reporting anatomical and functional observations, and it requires an explanation. We argue that a similar circuit structure in all cerebellar areas may carry out various operations using a common computational scheme. On the basis of a broad review of anatomical data, it is conceivable that the different roles of the cerebellum lie in the specific connectivity of the cerebellar modules, with motor, cognitive, and emotional functions (at least partially) segregated into different cerebro-cerebellar loops. We here develop a conceptual and operational framework based on multiple interconnected levels (a *meta-levels hypothesis*): from cellular/molecular to network mechanisms leading to generation of computational primitives, thence to high-level cognitive/emotional processing, and finally to the sphere of mental function and dysfunction. The main concept explored is that of intimate interplay between timing and learning (reminiscent of the “timing and learning machine” capabilities long attributed to the cerebellum), which reverberates from cellular to circuit mechanisms. Subsequently, integration within large-scale brain loops could generate the disparate cognitive/emotional and mental functions in which the cerebellum has been implicated. We propose, therefore, that the cerebellum operates as a general-purpose co-processor, whose effects depend on the specific brain centers to which individual modules are connected. Abnormal functioning in these loops could eventually contribute to the pathogenesis of major brain pathologies including not just ataxia but also dyslexia, autism, schizophrenia, and depression.

## Introduction

The cerebellum is classically thought to control movement coordination (Flourens, 1824; Luciani, 1891) and motor learning (Marr, 1969; Albus, 1972) but recent experimental evidence suggests that it may also play a key role in cognition and emotion (Schmahmann, 2004; Schmahmann and Caplan, 2006; Ito, 2008)^1^. This clearly raises broader questions: how can the same circuit cope with so many different tasks? Is signal processing in the cerebellar circuits always based on the same computational scheme? Is it conceivable that what underlies the different roles of the cerebellum is the specific connectivity of cerebellar modules, rather than specific microcircuit properties? In order to answer these questions, here we propose a conceptual and operational framework, or *meta-levels hypothesis*, based on four levels: (1) cellular/molecular, (2) network, primitives of circuit processing, (3) high-level cognitive/emotional processing, and (4) mental processing. We first review neuroanatomical, neuropsychological, neuropsychiatric, and neuroimaging studies in order to elucidate how the cerebellum might take part in cognitive and emotional functions, and how cerebellar damage could determine neurological and neuropsychiatric disorders. We then argue that the cerebellum carries out basic computational functions, timing, and learning, applicable in different cases. The cerebellum has been reported to assist brain operations by providing accurate timing of multiple series of signals coming from the cerebral cortex and the sensory systems [reviewed in Bower (1997, 2002); Jacobson et al. (2008); D'Angelo and De Zeeuw (2009); D'Angelo et al. (2009, 2010); D'Angelo (2010a,b, 2011); De Zeeuw et al. (2011)]. This could underlie the implementation of processes like sensory prediction, novelty detection, error detection, time matching, and sequence ordering (Ivry and Baldo, 1992; Ivry et al., 2002; Ghajar and Ivry, 2009). This multi-dimensional computation would allow the same circuit to contribute to functions as diverse as voluntary movement (a cognitive process, after all) and thought, provided that appropriate connections with different cortical and subcortical centers were established and that communication between these centers occurred over the appropriate frequency bands and using compatible codes (Ito, 1993, 2008; D'Angelo, 2011). We propose, therefore, that the cerebellum operates as a general co-processor, whose effect depends on the centers to which different modules are connected, affecting cognitive functions as well as sensory-motor processing.

## Brain processing and the cerebellum

The cerebellum has long been linked to the concept of motor control but now several observations indicate that it is also involved in cognitive/emotional processing. This extension of its role raises a key question: is there formal similarity between these two types of processing? A critical observation, in this regard, is that cognitive and sensory-motor processing should not, in principle, be very different. This prevents from a serious computational paradox: if the two processes were different, they may use different coding strategies. But then, how could the basic neuronal circuit of the cerebellum, which appears to be invariant across different areas, be able to process different signal codes? This would violate the idea that the cerebellum develops a single general algorithm. Indeed, different sections of the cerebral cortex (sensory, motor, and associative in nature) communicate with each other as well as with various cerebellar areas and so the neural codes are likely to be homogeneous. As a corollary of this, it is well-documented that motor planning means predicting the sensory consequences of a motor act: a motor plan is coded in terms of an anticipated sensory state (Blakemore et al., 1998a,b). This is akin to the general hypothesis of the “*prediction imperative*” that needs to be satisfied in order to allow brain processing (Llinás and Roy, 2009). Prediction processes are normally performed by “forward controllers,” which use internal memory to represent the system state (Diedrichsen et al., 2010; Shadmehr and Mussa-Ivaldi, 2012).

### Is the cerebellum a generalized forward controller?

On the basis of studies of the vestibulo-ocular reflex (VOR), eye-blink conditioning, and saccadic eye movements, and the fundamental theoretical concepts of motor learning (Marr, 1969), the cerebellum has been suggested to provide forward models of the motor system. These forward models can predict the posture or motion of body parts following a motor command and, by a further transformation, predict the sensory consequences of actions (Miall and Reckess, 2002). More precisely, a copy of motor commands generated by the motor cortex (*efference copy*) is sent to the cerebellum, which uses its internal forward model to predict their sensory consequences (*corollary discharge*). The sensory predictions are then compared to actual sensory feedback (Wolpert et al., 1998): in the presence of errors (or novelty, i.e., deviations from prediction), the cerebellum emits corrective signals. A fully characterized example of generation of predictions by cerebellar circuits is provided by electro-perception in weakly electric fishes, in which a cerebellar-like structure compares the expected electric field generated by the fish with the actual electric field sensed by the electroreceptors, thus gaining information on the structure of the environment through the changes that this latter has caused in the field itself (Bell et al., 2008).

In the presence of persistent deviations from prediction the cerebellum learns to modify the forward model itself. Learning appears to occur through two distinct processes, one faster and more labile, involving the cerebellar forward controller, the other, which may at least partly reside outside the cerebellum, slower and consolidated (Shadmehr and Mussa-Ivaldi, 2012). In fact, the cerebellar cortex is thought to process the faster component of memory, while the deep cerebellar nuclei may elaborate its slower component (Medina and Mauk, 2000). The cerebellum is thought to share its “predictor function” with the parietal lobes, in such a way that these two structures might work in parallel (Blakemore and Sirigu, 2003): the cerebellum as a whole is likely to generate faster but unconscious predictions, while the parietal lobes probably generate slower but conscious ones.

Given the anatomical connections of the cerebellum with associative areas (see below) and the similarity of motor planning and cognitive processing, it seems logical to generalize the forward controller role of the cerebellum to cognition. Indeed, Ito (2005) hypothesized that the cerebellum could operate as a generalized forward controller regulating cognition as well as sensory-motor control^2^.

### The forward controller and mental activity

The fundamental postulate about brain/mind functioning is that the brain generates a virtual reality (Churchland and Sejinowski, 1992; Churchland, 1998; Llinas and Paré, 1998), probably conferring an evolutionary advantage by predicting possible environmental configurations and allowing symbolic representation and communication. Several observations show that perception is not a copy of the external or internal energy patterns, but rather a mental elaboration endowed with quality and deformed by imperfect receptor sampling, adaptation, memories, and emotions. This makes conscious perception unique and subjective. At this point, one may speculate on how the cerebellum, being deeply interconnected with the cerebral cortex, might be involved in processing conscious percepts. We propose a somewhat provocative reflection.

A first issue is that whereas reality is perceived as *instantaneous*, computation in neurons and synapses actually takes time and the cerebral cortex needs hundreds of milliseconds to generate a conscious percept. This delay, in addition to violating the idea of the instantaneity of subjective perception, is far too long to allow movement and thought to be controlled in a purposeful, dynamic, and interactive manner. Therefore, the virtual reality generated by the brain has to be “anticipatory” and to occur somehow in advance of the elaboration of objective reality based on cortical processing of sensory signals. This anticipatory process may be based on the use of previous information and memory on various time scales, as would occur in a *forward controller*, which is exactly what the cerebellum is thought to be. A second issue is that reality is perceived as *continuous*, even though computational cycles during cerebro-cortical cognitive processing actually last about 25 ms (a γ-band cycle) and longer cycles about 100 ms (a θ-band cycle) (Buzsaki, 2006). The cerebellum, by exerting millisecond control of its output spikes, may help to maintain the *fast continuity* required for spatiotemporal integration of conscious percepts.

Thus, the fact that the cerebellum does not, clinically, appear to be needed to generate consciousness (Tononi and Edelman, 1998) does not mean that it is extraneous to the mechanisms controlling the relationship between objective reality and internal representation. Indeed, functional activation of the cerebellum has been revealed in relation to the conscious representation of time in tasks using internal memories (Addis et al., 2009; Nyberg et al., 2010; Szpunar, 2010, 2011). It should be noted at this point that one main theory on the working of the cerebellum is that it acts as a “comparator of intentionality with execution,” which is precisely what the whole brain continuously does in order to relate neuronal activity to the world. On this basis, we conclude that it can hardly be considered surprising that the cerebellum takes part in cognition and emotion, that it can influence attention and intelligence (Cotterill, 2001), and that its dysfunction can affect “internal coherence” in dissociative diseases.

## The extended cerebro-cerebellar loops

**Figure 1:**
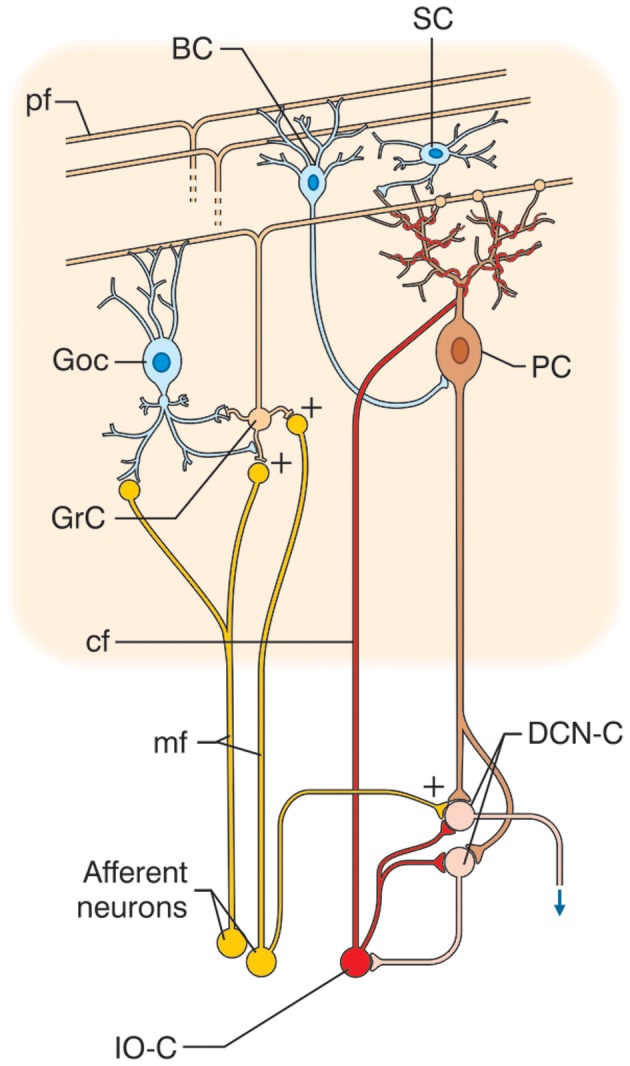
**Schematic representation of the cerebellar circuit.** The cerebellar circuit consists of cortical and subcortical sections. At subcortical level, the *afferent fibers* activate DCN cells (DCN-C) and IO cells (IO-C). The DCN emits the output and at the same time inhibits the IO. In the cerebellar cortex, there are different types of neurons including granule cells (GrC), Golgi cells (GoC), Purkinje cells (PC), stellate and basket cells (SC, BC), Lugaro cells, and unipolar brush cells (not shown). The two main inputs are represented by mossy fibers (mf) originating in various brain stem and spinal cord nuclei, and by climbing fibers (cf) originating from the IO. Signals conveyed through the mossy fibers diverge to DCN and activate the granular layer (containing GrC and GoC). The ascending axon of the GrC bifurcates in the molecular layer (containing PC, SC, and BC) forming the parallel fibers (pf). The cerebellar cortical circuit is organized as a feedforward excitatory chain assisted by inhibitory loops: mfs excite GrCs, which activate all the other cortical elements. In the granular layer, inhibition is provided by GoC, in the molecular layer by SC and BC. Finally, PC inhibit DCN. The IO, which is also activated by brain stem and spinal cord nuclei, controls PC activity though a single powerful synapse. Thus, the whole system can be seen as a complex mechanism controlling the DCN output.

The cerebellar cortex has, from the earliest studies, always been reported to have a similar structure in all its sections, and its circuit to show a regular “lattice”-like organization (Eccles et al., 1967) (Figure 1). The cerebellar circuit can be schematically described as follows: mossy fibers activate granule and Golgi cells in the granular layer. Granule cells emit parallel fibers and activate all the other neurons in the cerebellar cortex. Golgi cells are doubly activated by mossy and parallel fibers providing feedforward and feedback inhibition to granule cells. The granular layer also contains other interneurons, namely, Lugaro cells and unipolar brush cells (only in the flocculo-nodular lobe). In the molecular layer, parallel fibers activate Purkinje cells and also stellate and basket cells, which in turn inhibit Purkinje cells. Purkinje cells are also activated by climbing fibers generated by the inferior olive. Purkinje cells in turn project to the deep-cerebellar nuclei. In this context, the *modules* and the *cerebello-thalamo-cerebro-cortical* circuits (CTCCs) can be considered the main structural elements.

### The cerebellar modular organization

**Figure 2:**
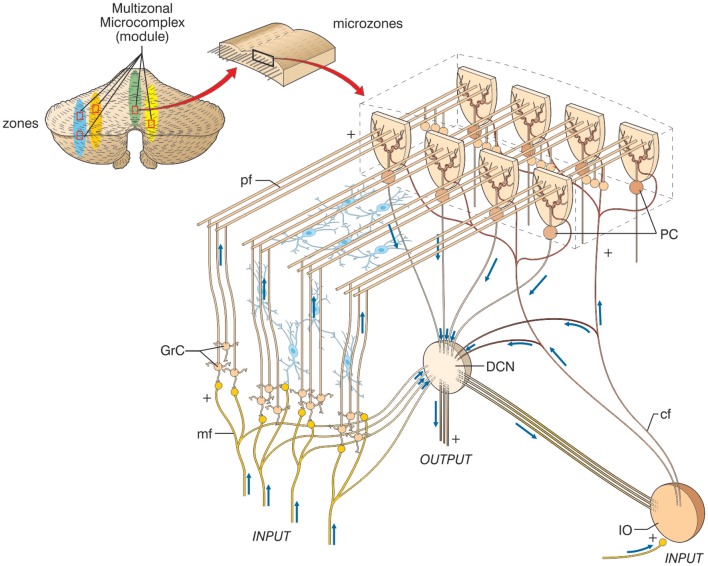
**The modular organization of the cerebellum.** The picture shows a flattened view of the cerebellum. Four ideal *zones* are shown in color, each one containing *microzones* forming a *multizonal microcomplex*. The microzones have the basic structure reported in the expansion on the right (same symbols as in Figure 1, inhibitory interneurons are overlaid in blue). A microzone is defined as a group of the order of 1000 Purkinje cells all having the same somatotopic receptive field. These Purkinje cells are arranged in a long, narrow strip, oriented perpendicular to the cortical folds, so that Purkinje cell dendrites are flattened in the same direction as the microzones extend and are crossed by parallel fibers at right angles. The climbing fibers branches (about 10) usually innervate Purkinje cells belonging to the same microzone and the olivary neurons generating such climbing fibers tend to be coupled by gap junctions. This helps synchronizing Purkinje cells within a microzone on a millisecond time scale. The Purkinje cells belonging to a microzone all send their axons to the same small cluster of output cells within the deep cerebellar nuclei. Finally, the axons of basket cells are much longer in the longitudinal direction than in the mediolateral direction (not shown), causing them to be confined largely to a single microzone. Thus, cellular interactions within a microzone are much stronger than those between different microzones.

Macroscopically, the cerebellum consists of a tightly folded layer of cortex with white matter beneath in which deep nuclei are embedded. At microscopic level, each part of the cortex consists of the same small set of neuronal elements, laid out according to a highly stereotyped geometry. At an intermediate level, the cerebellum and its auxiliary structures can be broken down into several hundred or thousand *microzones* or *microcompartments*, which are thought to represent effective cerebellar functional units (Figure 2). These can be further differentiated into *stripes*, *zones*, and *multizonal microcomplexes*, which are effective functional *modules* (Andersson and Oscarsson, 1978; Apps and Garwicz, 2005; Apps and Hawkes, 2009)^3^.

A module is a conglomerate of several, non-adjacent parasagittal bands of Purkinje cells projecting to specific areas of deep cerebellar nuclei and gating segregated projections from the inferior olive (Cerminara, 2010; Oberdick and Sillitoe, 2011; Ruigrok, 2010). Likewise, the mossy fibers projecting to a certain group of Purkinje cells through the granular layer also project to the same deep cerebellar nucleus neuron receiving input from those Purkinje cells (Ito, 1984; Pijpers et al., 2006; Voogd, 2010). The modules have almost segregated inputs, since climbing fibers bifurcate on the parasagittal plane to as many as 10 not necessarily adjacent Purkinje cells (mossy fiber bifurcations spread across both planes). Thus, the majority of connections between neurons and interneurons in the cerebellar cortex occur within individual modules. The connections between modules occur almost exclusively via parallel fibers, which contact Purkinje cells and the other inhibitory interneurons (Lainé and Axelrad, 1998; Dieudonné and Dumoulin, 2000; Dean et al., 2004).

The modules have a very similar if not identical structure and do not show major differences in their neuronal properties, even though some variants have been reported. One of these concerns the vestibulocerebellum, which contains an additional cell type, the unipolar brush cell (Mugnaini et al., 2011), and may exhibit more sustained discharges to Purkinje cells (Kim et al., 2011). Another peculiar aspect is glycine feedback from the lateral cerebellar nuclei, which is sent only to the hemispheres and not to the vermis (Uusisaari and Knopfel, 2012). Finally, evident organization of genetic markers along the sagittal plane leads to a further “biochemical” compartmentalization^3^. These local properties do not undermine the general concept of a unified cerebellar computational algorithm, but they may bias certain modules toward specific functional states, as is thought to occur in other brain circuits in relation to neuromodulators and neuropeptides (e.g., LeBeau et al., 2005).

The cerebellar circuit appears to be organized in a feed-forward manner, with information passing through the cortex without recurrent loops and with limited intermodular connectivity. This is in apparent contrast with the cerebral cortex, which shows zonal differences in thickness, in the proportion of granular and pyramidal neurons, in intracortical connectivity, in neuronal subtypes and spine distribution (Elston and DeFelipe, 2002; Douglas and Martin, 2004; Lubke and Feldmeyer, 2007). Moreover, while there is poor intermodular connectivity in the cerebellum, the cerebral cortex shows strong intercolumnar connectivity [the relevance of which has been commented above (Tononi and Edelman, 1998)]. Clearly, the different anatomo-functional organization of the two cortices implies different computational strategies. However, since the two cortices are deeply interconnected through serial parallel loops, the product of cerebro-cortical elaborations is continuously relayed to specific modules of the cerebellar cortex. Thus, in addition to the need to understand how cerebral and cerebellar cortical modules operate, it is essential to look in more detail at this interconnection of the two structures.

### Cerebello-thalamo-cerebro-cortical circuits (CTCCs)

**Figure 3:**
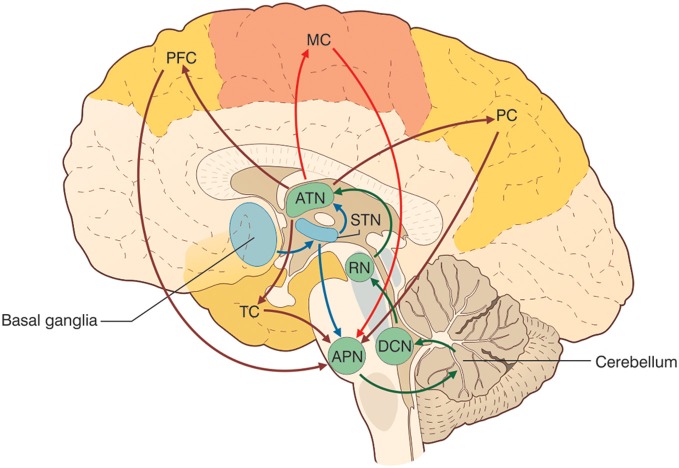
**The cerebello-thalamo-cerebro-cortical circuits (CTCCs).** The figure represents schematically the bidirectional connectivity between the cerebellum and the telencephalon, in particular with the cereberal cortex. Telencephalic projections from the cortex and basal ganglia (through the subthalamic nucleus, STN) and limbic areas are relayed to the cerebellum through the anterior pontine nuclei (APN). The cerebellum in turn sends its output through the deep cerebellar nuclei (DCN), red nucleus (RN), and anterior thalamic nucleus (ATN) to various telencephalic areas including the motor cortex (MC), the prefrontal cortex (PFC), the parietal cortex (PC), and the temporal cortex (TC). These connections, which are supported by anatomical and functional data, forming several bidirectional cerebello-thalamo-cerebro-cortical circuits (CTCCs).

There is growing evidence that the *CTCCs* include several afferent and efferent cerebral cortical areas of a motor, sensory, or associative nature (Figure 3) (Strick et al., 2009). Most cerebro-cerebellar *afferent* projections pass through the basal (anterior or ventral) pontine nuclei and intermediate cerebellar peduncle, while most cerebello-cerebral *efferent* projections pass through dentate and ventrolateral (VL) thalamic nuclei. Some of these loops are here considered in more detail in relation to sensory-motor and cognitive-emotional functions: the *motor and somatosensory loops* (including those involved in oculomotor control), the *parietal loops*, the *prefrontal loops*, the *oculomotor loops*, and the loops formed with the basal ganglia and the limbic system. Cerebello-cerebral loops are highly segregated (Habas et al., 2009; Krienen and Buckner, 2009) and form complex interconnections also with the basal ganglia and subcortical areas. Interestingly, during phylogenesis, the cerebellar hemispheres evolve in parallel with the associative rather than the motor or sensory areas, which supports the progressive involvement of the cerebellum in cognitive processing.

#### Motor and somatosensory loops

The cerebellum projects both to motor and somatosensory areas. The output to the primary motor area (M1) is conveyed through the VL thalamic nuclei projecting to layers IV and V, while outputs to the primary somatosensory area (S1) pass through the intralaminar nuclei projecting to intragranular and superficial layers (Molinari et al., 2002). Through these projections to MI, the cerebellum can modulate motor cortex excitability in relation to the incoming sensory input (Luft et al., 2005). The cerebellum is also interconnected with premotor (Dum and Strick, 2003) and supplementary motor areas (Rouiller et al., 1994) involved in movement planning. Interestingly, transcranial magnetic stimulation (TMS) of the lateral cerebellum can strongly affect the contralateral cerebral motor cortex (Oliveri et al., 2005). Cerebellar TMS regulates the functional connectivity between Purkinje cells and deep cerebellar nuclei, modifying the excitability of interconnected motor areas, as shown by changes in motor-evoked potential amplitude and in short and long intracortical inhibition (Koch et al., 2009a).

#### Parietal loops

The cerebellum is closely connected with the parietal lobes. The cerebellum sends input to area 7b of the inferior parietal lobe, in particular to the anterior intraparietal (AIP) area, through VL thalamic nuclei (Clower et al., 2001). AIP neurons are activated in response to the sight of an object, as well as to the act of grasping it, in reach-to-grasp arm movements (Tunik et al., 2005), and in the creation of crossmodal sensorial representations of objects (Grefkes et al., 2002). The cerebellar input to the AIP passes through a specific “output channel” of the dentate nucleus. The cerebellar-VL thalamic inputs to motor and premotor areas send secondary afferents to the AIP (Clower et al., 2005). The cerebellum also targets other parietal regions, namely the ventral lateral intraparietal area (vLIP) and medial intraparietal area (MIP) (Prevosto et al., 2010). Importantly, vLIP neurons can represent salient visual stimuli and are important for visual attentional control (Kusunoki et al., 2000), while the MIP is crucial for visual-motor coordinate transformation (Grefkes et al., 2004). In humans, the AIP is also connected to the ventral premotor cortex, while theMIP shows relatively strong projections to parahippocampal regions (Rushworth et al., 2006) forming complex loops involving multiple cortical areas, the thalamus, the cerebellum, and the basal ganglia.

#### Prefrontal loops

The cerebellum is reciprocally connected, through the thalamus (Middleton and Strick, 2001), with the medial prefrontal cortex (MPFC) (Watson et al., 2009), the dorsolateral prefrontal cortex (DLPFC) (Kelly and Strick, 2003), and the anterior prefrontal cortex (APFC) (Krienen and Buckner, 2009). The MPFC is important in saccadic movements and cognitive control (Ridderinkhof et al., 2004) and is strongly involved in determining behavior on the basis of expectations (Amodio and Frith, 2006). Moreover, this cortical area plays a key role in fear extinction processes (Morgan et al., 1993; Milad and Quirk, 2002). The DLPFC is particularly important in working memory control (Petrides, 2000), mental preparation of imminent actions (Pochon et al., 2001), and procedural learning (Pascual-Leone et al., 1996) and its functional alteration is involved in major psychoses (Weinberger et al., 1986, 1988; Dolan et al., 1993). The APFC is less understood (Ramnani and Owen, 2004) but its main function could be that of integrating multiple distinct cognitive processes during goal-directed complex behaviors.

#### Temporal loops

The exact nature of the connections between temporal areas—including the hippocampus and amygdala—and the cerebellum is still unclear. However, some studies have shown that the temporal cortex makes a “negligible” contribution to the corticopontine fiber tract (both in humans and in macaque monkeys) (Ramnani et al., 2006). This probably means that the cerebellum is unlikely to receive strong *direct* afferents from temporal areas. On the other hand, cerebellar fastigial nuclei seem to project to several temporal areas, like the hippocampus and amygdala (at least in monkeys and cats) (Heath and Harper, 1974). Accordingly, a recent human fMRI resting-state study found significant functional connectivity between the bilateral anterior inferior cerebellum and bilateral hippocampus and temporal lobes (He and Zang, 2004). Furthermore, dynamic causal modeling proved that, during a rhyming judgment task, the cerebellum and the lateral anterior temporal lobe are strongly and bidirectionally interconnected (Booth et al., 2007). More extensive studies are clearly required in order to elucidate the pattern of connectivity between the cerebellum and temporal areas; however, it seems reasonable to speculate that there exists some kind of functional interplay between these two structures.

#### Oculomotor loops

The cerebellum is also deeply involved in oculomotor regulation, which involves several cortical and subcortical areas participating in automatic and cognitive control processes. Besides the VOR, to which the cerebellar flocculo-nodular lobe is specifically devoted, the cerebellum is involved in the control of saccadic and smooth pursuit eye movements (Alahyane et al., 2008; Colnaghi et al., 2010; Panouillères et al., 2011). Both the lateral and posterior cerebellum, mainly the vermis, are involved in the control of ocular saccades (Robinson et al., 1993; Hashimoto and Ohtsuka, 1995; Goffart et al., 2003). The lateral cerebellum and the vermisare also involved in controlling the precision and velocity of smooth pursuit movements (Takagi et al., 1999). Saccades and pursuit, used in order to execute different cognitive-perceptual tasks (basically, saccades are required when searching for a static target, while pursuit is needed to track moving targets), are thought to be different outcomes of a single sensory-motor process aimed at orienting the visual axis (Xivry and Lefèvre, 2007). The cerebellum and the fastigial oculomotor region have been shown to play a major role both in controlling the execution of saccades and in elaborating the visuospatial information concerning the target (Tilikete et al., 2006; Guerrasio et al., 2009). The oculomotor system comprises different areas. The retina projects to the superior colliculus (Lefèvre et al., 1998), which, in turn, sends afferents to the cerebellum and the lateral intraparietal area (LIP). The LIP is connected with the frontal eye field (FEF) and the basal ganglia and superior colliculus gate input from the FEF to the LIP (Straube and Buttner, 2007). A recent Diffusion Tensor Imaging (DTI) study (Doron et al., 2010) showed the cerebellum to be strongly connected with the precentral gyrus and the superior frontal gyrus, which take part in motor and oculomotor processes as well as the processing of spatial working memory (Boisgueheneuc et al., 2006). The cerebellum has thus been shown to be deeply integrated in processes controlling both the motor and cognitive components of eye movements.

#### Loops formed with the basal ganglia and limbic system

The cerebellum has recently been shown to form bidirectional connections with the basal ganglia. The cerebellum-basal ganglia pathway starts from the dentate nucleus, goes through the thalamus and reaches the striatum; the basal ganglia-cerebellum pathway starts from the subthalamic nucleus and ends in the cerebellar cortex, passing through the pontine nuclei (Bostan and Strick, 2010; Bartolo et al., 2011). The cerebellum is also thought to be connected with the limbic system, although few anatomical studies are available. Low-frequency stimulation of the cerebellar fastigial nucleus has an anti-epileptogenic effect when seizures are induced by amygdaloid kindling (Wang et al., 2008) and there exists evidence suggesting that the cerebellum may be connected with the amygdala, hippocampus, and septal nuclei (Snider and Maiti, 1976). The cerebellum is also connected with the hypothalamus (Haines et al., 1990) and, as indicated above, with limbic cortices like the DLPFC.

### Functional activation of cerebro-cerebellar loops

One of the greatest recent achievements of neurophysiology has been to open a window on the mechanisms governing cognitive and emotional functions. Techniques like fMRI and Magnetoencefalography (MEG) have proved fundamental in this respect, since they provide information on the localization and correlation of active areas during controlled behavioral tasks. Moreover, the use of TMS has made it possible to intervene selectively on the CTCCs (by directly exciting or inhibiting or by modifying synaptic plasticity). In this way, neuroanatomy can be turned into functional connectivity, linking circuit organization with system functions and behavior, so that mental activity and major mental disorders can be explored on a physiological basis. In parallel with these developments, understanding of cerebellar functions is also improving greatly.

The close relationship between the cerebellum and cerebral cortex was first revealed by crossed cerebellar diaschisis, a reduction in metabolism and blood flow in the cerebellar hemisphere contralateral to a cerebral lesion (Beldarrain et al., 1997). Detailed investigations have since provided structural and functional evidence (see also below) of multiple cerebro-cerebellar loops processing, in concert, sensory-motor and emotional/cognitive tasks. In fMRI studies, cognitive and motor functions in human CTCCs appear segregated (Salmi et al., 2010). A non-verbal auditory working memory task was found to be associated with enhanced brain activity in the parietal, dorsal premotor, and lateral prefrontal cortices and in lobules VII–VIII of the posterior cerebellum. A sensory-motor control task activated the motor/somatosensory, medial prefrontal, and posterior cingulate cortices, and lobules V/VI of the anterior cerebellum. A purely cognitive task activated fronto-parietal cerebro-cortical areas and crus I/II in the lateral cerebellum. The tracts between the cerebral and the cerebellar areas exhibiting cognitive and sensory-motor activity are mainly projected via segregated pontine (input) and thalamic (output) nuclei. For example, crus I/II in the lateral cerebellum is linked with the DLPFC and is activated during cognitive tasks, whereas the anterior cerebellar lobe is not.

Functional imaging studies have helped to confirm the relationship between the specific activation of the latero-posterior lobe and cognitive processes during cerebellar damage, often associated with a frontal-like syndrome (see below) with memory deficits and aphasia, thought dysmetria, and incoordination between mental processing and motor execution (Arriada-Mendicoa et al., 1999). Moreover, malformations of or damage to the cerebellar vermis are commonly linked to affective alterations (Schmahmann and Sherman, 1998; Tavano and Borgatti, 2010). These observations support the view that cognitive/emotional and motor functions are at least partially segregated in the cerebellum, with cognitive functions localized in the lateral-posterior cerebellum.

## From motor control to cognition and emotion

Neurology classically considers the cerebellum in relation to *ataxia*, i.e., the motor consequences of cerebellar damage. Ataxia (from the Greekα–ταξισ, meaning “lack of order”) is a neuropathological state consisting of gross lack of coordination of muscle movements. It is caused by dysfunction of those parts of the nervous system that coordinate movement and it includes forms of cerebellar, sensory, and vestibular origin. Cerebellar ataxia is expressed through a variety of elementary neurological deficits, such as antagonist hypotonia, asynergy, dysmetria, dyschronometria, and dysdiadochokinesia. How and where these abnormalities manifest themselves depends on which cerebellar structures have been damaged and whether the lesion is bilateral or unilateral. In very general terms, we can observe three main groups of symptoms^4^:

- impairment of body balance (Romberg test) and of eye movement control (saccade alterations, nystagmus) due to specific dysfunction of the vestibulocerebellum;
- impairment of gait (wide-based, “drunken sailor” gait, characterized by uncertain starting and stopping, lateral deviations, and uneven steps) due to dysfunction of the spinocerebellum;
- difficulty executing voluntary, planned movements due to impairment of the cerebro-cerebellum. Disturbances include intention tremor (coarse trembling, accentuated on the execution of voluntary movements, possibly involving the head and eyes as well as the limbs and torso), peculiar writing abnormalities (large, uneven letters, irregular underlining), and a peculiar pattern of dysarthria (slurred speech, sometimes characterized by explosive variations in voice intensity despite a regular rhythm).

Quite apart from their undisputed clinical importance, these observations lend support to the idea that different motor functions are localized in specific cerebro-cerebellar loops and that the lateral cerebellum is involved, through cerebro-cerebellar loops, in the cognitive components of movement planning. In addition, on careful analysis, patients with focal cerebellar lesions have also been found to show cognitive-affective alterations (Schmahmann and Sherman, 1998) constituting a picture that might be called *dysmetria of thought*. The concept of “dysmetria of thought” or “cognitive dysmetria” has been proposed as a unitary neurocognitive framework of reference for schizophrenia symptoms [(Andreasen et al., 1998), see below] and involves a neural network with the main nodes in the prefrontal cortex (PFC), thalamus, and cerebellum. Cognitive dysmetria comprises:

- impairment of executive functions, such as planning, set-shifting, abstract reasoning, working memory, and verbal fluency;
- difficulties with spatial cognition, both in visuospatial organization and visuospatial working memory;
- personality change, with blunting of affect and/or disinhibited and inappropriate behavior;
- language deficits including agrammatism, dysprosodia, and mild anomia.

This constellation of symptoms, which is reminiscent of a prefrontal syndrome (Schmahmann, 2004; Schweizer et al., 2007), is called *cerebellar cognitive affective syndrome*. Clearly these symptoms are not exclusive to cerebellar damage; indeed, the aforementioned cognitive and affective alterations can also be found in patients with disorders of the cortical associative areas (especially prefrontal) and paralimbic areas, or with disorders of the subcortical areas to which the former are connected. It would be safe to say that these symptoms involve the whole CTCC loop. Anatomically, lesions of the posterior lobe are associated, in particular, with cognitive symptoms, while lesions of the vermis are consistently observed in patients with pronounced affective alterations. The anterior lobe seems to be less involved in the generation of these cognitive and behavioral deficits, while anterior lobe lesions are well-known to cause motor ataxia (Diener and Dichgans, 1992) (Figure 3). Functional neuroimaging studies have consistently shown: (1) activation in the anterior lobe during motor learning and classical conditioning, (2) activation of the posterior lobe during several kinds of purely cognitive tests of executive functions (cognitive planning, set-shifting, working memory), language (verbal memory tasks, verb for noun substitution, synonym generation), mental imagery, and sensory discrimination, (3) activation of the vermal region during tests evaluating emotional modulation. Finally, (4) abnormal activation of the cerebellar vermis and posterior lobe has been observed in several primary psychiatric disorders, most notably schizophrenia, autism, and dyslexia, further discussed below.

### The extended coordinating and predicting action of the cerebellum

The cerebellum is assumed to contribute to sensory-motor processing in an automatic manner. After having received, analyzed, and recognized a sensory or a motor pattern (as a prediction of a future sensory state), the cerebellum produces gain and phase corrections that make it possible to regulate the force and activation of large sets of muscles^5^. The predicted and actual patterns are then compared; this is followed by the provision of appropriate correction sand thus the generation of movement *coordination*. As an extension of this, patterns coming from various cerebro-cortical areas can be processed, allowing the “coordination” of higher cognitive functions. Once activated, the CTCC loops could be used not just for *automatic* but also for *controlled* functions. These can be set in the more general framework of *cognitive control* and *executive function*^6^.

The cerebellum may take part in *cognitive control* by regulating executive functions, which it could do by manipulating different “objects.” These can be considered parts of a set of virtual representations, given that they may be purely symbolic (e.g., thoughts) or applied to symbolic expression (e.g., speech) or voluntary movement (which, after all, is based on a virtual representation of its sensory consequences—see above). The cerebellum then integrates these multiple internal representations (of a motor, sensory, or cognitive/emotional nature) with external stimuli and with voluntary (or self-generated) responses. Indeed, cognitive dysmetria, which is the loss of these functions, is characterized by difficulty in prioritizing, processing, and coordinating responses to incoming information (Andreasen et al., 1996; Crespo-Facorro et al., 1999). Importantly, the involvement of the cerebellum in *executive functions* becomes more prominent as the complexity of these functions increases (Gottwald et al., 2004). Deficits in semantic and phonemic fluency and poor performances reported in some memory tasks can be traced back to a deficit in executive functions. Moreover, performance on “basic” attentional tasks (e.g., Go/NoGo) is substantially normal, but performance on “high level” attentional tasks (e.g., the “divided attention” paradigm, where subjects have to respond simultaneously to multiple cognitive tasks) is impaired (Baddeley et al., 1984; Craik et al., 1996). Finally, patients with right-sided lesions are more impaired than those with left-sided lesions. This supports the idea of lateralization of cerebellar functions, with verbal deficits mostly occurring in the presence of right cerebellar lesions and visuospatial deficits tending to occur in left cerebellar lesions. Clearly, this lateralization replicates the division of cognitive competences between the two cerebral hemispheres, with which the cerebellum is cross-connected via the pontine nuclei and thalamus.

A similar role of the cerebellum in prioritizing, processing, and coordinating responses to incoming information could underlie cerebellar control of emotional experience^7^. Lesions of the cerebellum interfere with affective expectations from a given behavioral context. This is evident in fear conditioning paradigms, in which the relationship between a conditioning stimulus and a frightening unconditioned stimulus can be precisely controlled (Sacchetti et al., 2005). Vermal lesions can decrease reactivity to frightening stimuli, probably by controlling the output to the hypothalamus, amygdala, hippocampus, septal nuclei, and nucleus accumbens. Likewise, neuroimaging studies show that the cerebellum and the anterior cingulate cortex (ACC) are strongly activated when a painful stimulus is expected after a given cue (Ploghaus et al., 2003). While the cerebellum builds up the expectation of pain, the ACC, which is strongly connected with the cerebellum, plays an important role in several neurocognitive mechanisms capable of modulating pain perception, mainly attention, expectation, and reappraisal (Wiech et al., 2008). Moreover, the cerebellum, together with the ACC and the insula, is strongly activated when perceiving pain in others (Jackson et al., 2005), and these same structures (together with the primary and secondary somatosensory cortices, putamen, and thalamus) have been found to show activation that is related to the intensity of pain (Coghill et al., 1999). Finally, the cerebellum may also regulate the quality of emotional experience (Turner et al., 2007). Patients with cerebellar stroke report reduced pleasant feelings in response to happiness-evoking stimuli (while unpleasant experience to frightening stimuli was substantially similar to that recorded in controls).

**Figure 4:**
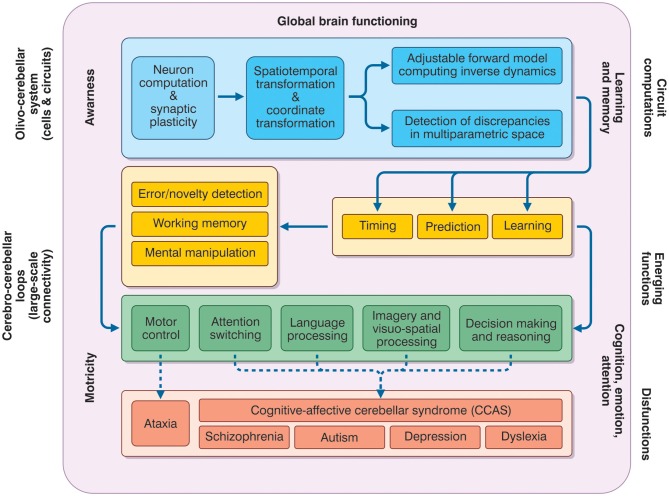
**The meta-levels of cerebellar activity.** The figure depicts the causal relationships between the functions that the cerebellum is thought to play at different operative levels (meta-levels hypothesis) and between these same functions and brain pathologies. The *neuron and network* level lays in the blue box and is normally investigate using electrophysiological and imaging techniques. These combined with mathematical models, allow to infer the *computational functions of the circuit* (forward model, various parameter transformation and detection of discrepancies between predicted and actual signal patterns). Once integrated into the large-sale connectivity of the CTCC loops, circuit computations lead to emerging functions. At low-level (yellow boxes), these include *learning, prediction, and timing* (cerebellar processing primitives), which can implement structured cerebellar operations including forms of *working memory, error/novelty detection, and mental object manipulation*. The low-level functions lay at the basis of more complex high-level functions (green box) including *motor control, attention switching, language processing, imagery and visuospatial processing, decision-making, and reasoning*. Major aspects of brain pathology (red box) can be predicted on the basis of the low- and high-level functions. Emerging functions and dysfunctions are usually investigated using non-invasive recordings (fMRI, DTI, TMS etc.), neuropsychological and clinical assessments. As a whole, the cerebellar function can contribute to global brain operations not just of motricity but also of learning and memory, cognition, emotion, attention, and even awareness.

The prefrontal cerebral cortex has classically been considered to be the main station exerting cognitive control and the limbic system cortices to be the ones primarily involved, together with amygdala and hippocampus, in affective control. Infact, signals processed in the cerebral cortex are continuously sent to subcortical structures, including the cerebellum, which then sends back to the cortex signals able to refine and control cerebro-cortical processing. This process resembles the control of movement planning occurring in the sensory-motor CTCC loops (Figure 4).

### Meta-levels of signal processing in CTCC loops

So far we have considered observations suggesting that the cerebellum, in addition to taking part in sensory-motor control, is also involved in cognitive/emotional functions. These observations are based on evidence of cerebellar activation during specific cognitive/emotional tasks and on the existence of connections between the cerebellum and relevant cerebro-cortical areas. Moreover, we have tried to make sense of all this by setting cerebellar activity within the general framework of brain functioning and cognitive control. But the question, now, is how can the cerebellum support these multiple operations? The basic hypothesis is that the cerebellum uses, throughout, the same circuit structure, and that different outcomes depend on the specific connections to different brain areas. This also implies that the same code is used for all the operations involving the cerebellum and that motor control and cognition/emotion have an equivalent structure at the level of spike coding.

On an operational level, in order to connect basic circuit functions with cognitive/emotional and mental processing, a series of meta-levels needs to be considered. Ideally, it should be possible, first, to demonstrate the connection between neighboring meta-levels, and thereafter to link the cellular/molecular mechanisms with cognitive/emotional processing and then with mental function and dysfunction.

1. *Cellular/molecular to circuit*. As regards the relationship between the cellular-molecular level and the circuit level of cerebellar operations, several specific hypotheses have been advanced, which are currently under investigation and have been discussed elsewhere (D'Angelo, 2011). The idea, basically, is that the cerebellum is able to exploit spike timing, neuronal dynamics and long-term synaptic plasticity in order to process incoming signals in the spatial, temporal, frequency, and phase domains. At circuit level, timing and plasticity in neurons and synapses can implement adaptable signal processing capabilities, which appear to be the prerequisites for the emergence of cerebellar processing (Hansel et al., 2001; D'Angelo and De Zeeuw, 2009). The outcome of circuit operations on cerebellar functions are themselves bound to signal timing and learning, in line with the original main theories of the cerebellum as a timing and learning device (Albus, 1972; Ivry and Keele, 1989). While connecting circuit operations to emergent behaviors is obviously a fundamental step in understanding how the cerebellum operates, cellular/molecular mechanisms pertain to a different realm and will not be covered here (D'Angelo, 2011).
2. *Processing primitives*. At a low-level of complexity, cerebellar circuit computations emerge in the operations of *timing, sensory prediction*, and *sequence learning*. These can be tested in simple experimental tasks and, once embedded in appropriate CTCCs and larger brain systems, may be regarded as a basis for explaining more complex sensory-motor cognitive-emotional operations.
3. *High-level cognitive processing*. The outcome of processing primitives, applied to complex behavioral operations and involving multiple interconnected brain areas, could lead to various high-level cognitive operations. These include *attention, language, working memory, visuospatial processing, imagery, reasoning, and decision-making*.
4. *Mental processing and psychiatric diseases*. At the highest level, cognitive/emotional functions can be integrated into mental processing. Dysfunction of the relative mechanisms emerges through complex pathological manifestations including *autism, schizophrenia, depression*, and *dyslexia*.

## The cerebellar processing primitives

Understanding how the cerebellum contributes to so many apparently disparate functions would be an enormous step forward as it would mean understanding the common *processing primitives* of the cerebellar circuit. The most plausible hypothesis is that the cerebellum has a predictive function, i.e., the ability to anticipate incoming information and thus to ensure that actions correctly anticipate changes in the environment (Moberget et al., 2008). This hypothesis has two parts. The *timing hypothesis* postulates that the cerebellum is critical for representing the temporal relationship between task-relevant events, while the *sensory prediction hypothesis* postulates that it is critical in generating expectancies regarding incoming information (Ivry et al., 2002). The two hypotheses are not mutually exclusive; rather, they seem to be set at two different hierarchical levels, with timing being more elementary than prediction. Indeed, whereas timing is merely the establishment of an ordered relationship between two elements, the ability to predict future patterns (as in *a forward controller*) requires, in addition, the ability to compare different incoming patterns and predict their consequences on the basis of internally stored memory. Computationally, timing requires only one processing line while sensory prediction requires several. In the Pellionisz and Llinas hypothesis (Pellionisz and Llinàs, 1982), this sensory prediction corresponds to a tensorial transformation in the spatiotemporal hyperspace of the cerebellar circuit. Finally, the cerebellum is likely to use internal memories to adapt its computational schemes. The meaning of cerebellar learning has been hotly debated, with controversy often arising over the role of long-term synaptic plasticity in motor learning. Nonetheless, compelling evidence suggests that learning helps to automate timing and sensory prediction with respect to specific motor and cognitive sequences.

### Timing

Motor coordination, which fails in cerebellar patients, is essentially a precise spatiotemporal sequence of movements of one or more body segments, which must show appropriate position, velocity, and acceleration. As Ivry underlines (Ivry, 2000), the cerebellum probably operates as an internal timing system providing a precise temporal representation for motor and non-motor tasks. Experiments of “irregularity detection,” measuring cortical mismatch-negativity, have indicated that the cerebellum selectively contributes to processing the temporal properties of stimuli (Ivry, 2000). With regard to time estimation ability (timing), a recent review (Koch et al., 2009b) showed that the cerebellum is crucial when normal subjects are required to estimate the passage of brief time intervals and when time is computed in relation to given salient events. In turn, circuits involving the striatum and substantia nigra, which project to the PFC, are mainly implicated in processing supra-second time intervals in relationship with various cognitive functions.

One critical issue in physics and biology is velocity estimation, a process that could occur in different locations in the brain, such as the thalamo-cortical circuit (Ahissar et al., 2000; Szwed et al., 2003). As the cerebellum is a dedicated space-time processor, it is to be expected that it is also involved in velocity estimation. Indeed, a recent fMRI study (O'Reilly et al., 2008) identified a region in the posterior cerebellum (lobule VII crus 1) that is selectively activated during velocity judgment tasks (prospective spatiotemporal model). Conversely, when perceptual judgments are based only on the spatial (direction) characteristics of an object, this specific area is not significantly activated. Moreover, the functional connectivity between the posterior cerebellum and the anterior putamen (bilaterally), which is involved in timing (Matell and Meck, 2004), is enhanced during the velocity judgment task, which is essentially perceptual, with an only minimal motor component.

### Prediction

As we have pointed out, the cerebellum has been considered to act as a forward controller (Miall and Reckess, 2002; Wolpert et al., 1998) implementing the contravariant transformations that are necessary in order to convert predictive sensory plans into motor representations. The involvement of the cerebellum is shown by the ability to predict the sensory consequences of one's own motor actions. Typically, in the absence of visual feedback, cerebellar patients have great difficulty in estimating the direction of pointing (Synofzik et al., 2008). The cerebellum signals discrepancies between predicted and actual sensory consequences of movements, triggering appropriate corrections. In a recent study, subjects were required to use their right hand to move a robotic arm; the motion of this arm determined the position of a second robotic arm, which made contact with subject's left palm. Computer-controlled delays were introduced between the movement of the right hand and the tactile stimulation on the left palm. Activity in the right lateral cerebellar cortex, measured with PET, showed a positive correlation with delay, i.e., with the time prediction error (Blakemore et al., 2001). This suggests that the cerebellum is less activated by a movement that generates a tactile stimulation than by a movement that does not (which signifies an error due to lack of sensorial feedback from the target). A similar phenomenon is seen with tickling, whose sensory effect is suppressed during self-stimulation (which signifies perfect cancellation of error) (Blakemore et al., 1998a). Accordingly, the somatosensory cortex is significantly more activated by an externally generated tactile stimulus than by a self-generated one, and the cerebellum has been shown to provide the signal needed to attenuate the sensory responses to self-generated tactile stimuli (Blakemore et al., 1998a, 1999; Blakemore and Sirigu, 2003).

### Learning

Another basic function of the cerebellum is sequence learning. In a scenario simulating the absence of coordination in ataxia, Shin and Ivry (2003) showed that patients with cerebellar lesions were not able to learn simultaneously presented spatial and temporal sequences (conversely, patients with Parkinson's disease were able to learn these sequences, but not the relationship between them).

Along the same lines, cognitive sequencing functions can be selectively damaged in patients with cerebellar lesions; for example, patients with left-side cerebellar lesions perform poorly on script sequences based on pictorial material and patients with right-side cerebellar lesions on script sequences requiring verbal elaboration (Leggio et al., 2008). These deficits were not correlated with general intelligence, or with general neuropsychological impairment. Furthermore, they were found both in patients with focal lesions and in subjects with degenerative cerebellar pathologies. It is noteworthy that when these patients were asked to order a set of cards representing several behavioral sequences, they were unable to work out the correct order, even though they could correctly describe and understand the meaning of the single cards. Interestingly, while cerebellar patients are not necessarily impaired in learning simple visual or spatial sequences, their ability to discriminate different durations of auditory stimuli is generally impaired. Indeed, learning sequences of auditory tones with different durations has been found to be rather difficult for cerebellar patients, even though the same patients can normally learn visual sequences and sequences of tones with different frequencies but not different durations (Frings et al., 2006; Ivry and Keele, 1989). These data are obviously consistent with the “timing hypothesis”; however, the impairment in script sequences could be related to more abstract cognitive processes and possibly to a lack of executive functions.

The role played by cerebellar structures in sequence learning depends on experience-related factors; in motor sequence learning tasks, the cerebellum shows prominent activation during early phases of learning; instead, after extended practice, the activation is located mainly at the level of the basal ganglia (Doyon et al., 2002). Notably, within the early phase of learning, the activation has been found to shift gradually from the cerebellar cortex to the deep cerebellar nuclei (Medina and Mauk, 2000; Shadmehr and Mussa-Ivaldi, 2012). Moreover, some researchers hypothesize that the cerebro-cerebellar loop is primarily involved in motor adaptation processes (e.g., adapting to environmental changes or perturbations), rather than in effective motor learning processes (e.g., learning new sequences of movements), which could be processed by cerebro-striatal circuits (Doyon et al., 2003; Debas et al., 2010). The cerebellum, coupled with the PFC, is particularly important in learning new visuomotor procedures by imitation (Petrosini, 2007) in the manner of *mirror neuron* effects. Finally, cerebellar damage can lead to severe impairment of non-motor associative learning independently of motor alteration (Drepper et al., 1999).

## The cerebellum and high-level cognitive processing

The timing, predictive, and learning properties of the cerebellum, once integrated within the circuits formed with the cerebral cortex, basal ganglia, and limbic system, can lead to control of more complex cognitive/emotional functions, including attention, language, memory, imagery, and reasoning.

### Attention

The cerebellar contribution to attentive functions has been revealed in several physiological and pathological conditions. Both autistic and cerebellar patients show a selective impairment in attention shifting from visual to auditory stimuli, although attention focusing is normal (Courchesne et al., 1994a). Moreover, the cerebellum, controlling the precision of saccades, probably plays an important role in orienting attention to a visual cue (especially in covert attention tasks). This role seems to be linked to procedural spatial learning functions, which are strongly related to the ability of the cerebellum to learn goal-directed trajectories, as recently supported by experimental results (Burguière et al., 2005) and computational modeling (Passot et al., 2009).

Indeed, patients with cerebellar lesions are able to correctly orient visual attention but their reaction times are rather slow (800 and 1200 ms) compared with those of normal control subjects (100 ms on average) (Townsend et al., 1999). Attention switching is reinforced when subjects have to reassign motor responses to different stimuli. In agreement with this “attentive hypothesis,” some cerebellar areas show significant activation, measured with fMRI, during early phases of skill learning (both for motor and non-motor skills) and during pure visual attention tasks (Allen et al., 1997).

One theory is that the primary role of attention is to generate time-based expectancies of sensory information (Ghajar and Ivry, 2009). Essentially the suggestion is that, the higher the level of attention, the lower the performance variability, because the subject is less likely to be distracted by irrelevant information. The authors observe that the cerebellum is constantly activated after an attentional cue, independently of actual execution of movements, and even if the preparation of a potential motor response may be required. Accordingly, the cerebellum is bilaterally activated when a cue precedes the beginning of a motor task, whilst the primary motor cortex is activated only—and mainly contralaterally—during the execution of the task itself (Cui et al., 2000). Furthermore, it has been shown that PFC-projecting zones of the cerebellum process the symbolic content of sensory cues (Balsters and Ramnani, 2008). Ghajar and Ivry argue that the cerebellum may be actively involved in an attentional network comprising mainly the PFC, the inferior parietal lobule, and the cerebellum itself. The specialized role of the cerebellum might be to help encode the precise timing of sensory predictions. Cerebellar predictive activity probably works in a time frame of 2.5 s, so that events that fall within this window can be considered temporally bound.

Thus, according to Ghajar and Ivry's hypothesis, the predictive function of the cerebellum may be seen as a defining trait of attention. However, we can speculate that, in many tasks, attention is not necessarily closely bound up with sensory anticipation. The execution of visual search and feature match tasks, for example, may not rely on anticipatory mechanisms and may not involve the cerebellum directly. Nevertheless, cerebellar patients can fail in tasks of this kind, too, because impaired ocular movement control may lead to incomplete exploration of stimuli.

### Language processing and verbal working memory

The cerebellum is deeply implicated in language, involving both motor and cognitive processing organized in the “phonological loop.” Cerebellar pathology impairs acquisition of motor skills and primary articulatory abilities and the resulting reduced articulation speed impairs working memory for verbal material, reducing sensitivity to the onset, rime, and phonemic structure of language. This impairment of the phonological loop, in turn, leads to difficulty in language acquisition and dyslexia (Nicolson et al., 2001b) (see below).

Cerebellar damage can result in impairment of verbal working memory (Justus et al., 2005). Cerebellar patients demonstrate a reduction of the “phonological similarity effect” (normal subjects show more difficulties in memorizing phonologically similar words than phonologically dissimilar ones). Desmond et al. (1997) attempted to clarify the difference between the cerebellar contribution to phonological “rehearsal” mechanisms and to proper verbal working memory processes. During simple letter repetition tasks under fMRI, specific areas of the posterior vermis (lobules VI and VIIA) and of the cerebellar hemispheres (left superior HVIIA, right HVI) were activated. The same areas were activated together with an additional part of the right cerebellar hemisphere (HVIIB) during a sequential verbal working memory task. It was hypothesized that (1) HVIIA and HVI activations represent input from the frontal lobes (which are connected with the articulatory control processes of verbal working memory) and that (2) HVIIB reflects input from temporal and parietal areas (which, in turn, are probably the key areas of the phonological store), and that the function of the cerebellum during verbal working memory tasks could be to compare the output of subvocal articulation with the content of the phonological store. The verbal working memory deficit in cerebellar subjects is specific and is, both “forward and backward,” independent of dysarthric symptoms, which suggests that the cerebellum is involved in the initial phonological encoding and, possibly, in strengthening memory traces (Ravizza et al., 2006). In normal subjects, single-pulse TMS delivered to the cerebellum during the encoding phase of a verbal working memory test does not affect the accuracy of the performance but lengthens the reaction times (Desmond et al., 2005). Clearly, the involvement of the cerebellum in linguistic processing reflects the role of this structure in timing, learning, prediction, and attention.

Cerebellar patients show poor performances on phonological verbal fluency tasks, but not on semantic verbal fluency tasks [(Leggio et al., 2000); but see Smet et al. (2007)], and therefore show a dissociation between their processing of phonological and semantic material. Patients with aright posterolateral cerebellar lesion are selectively impaired in verb-noun associations (Gebhart et al., 2002). This impairment is not observed when the task is to associate verbs with visual stimuli (pictures of objects) (Richter et al., 2004). It should be noted that cerebellar patients, unlike patients with Parkinson's disease, are normally able to perform category learning tasks (Maddox et al., 2005). When listening to disyllabic stimuli, subjects with bilateral cerebellar pathology do not show the phoneme-boundary effect generally shown by neurologically normal subjects. This may be due to their impaired ability to discriminate between intervals of different duration (Ackermann et al., 1997). Clinical studies also suggest that cerebellar pathology can play a causal role in prefrontal aphasic symptoms (Marien et al., 1996). Moreover, cerebellar activity switches hemispheres (from right to left) according to recruitment of right PFC, during linguistic tasks, in aphasia following a stroke of left cerebral hemisphere (Connor et al., 2006).

The (right) cerebellum is strongly activated during semantic disambiguation tasks (Bedny et al., 2008) and, bilaterally, during lexical decision tasks with semantic priming (Rissman et al., 2003). The cerebellum is activated during different kinds of verb-noun association tasks (Seger et al., 2000). Also, the cerebellum is strongly activated by semantic discrimination tasks and the intensity of the activation correlates positively with the difficulty of the task (Xiang et al., 2003). Finally, it should be noted that cerebellar theta-burst stimulation with TMS has been shown to selectively enhance associative priming, while semantic priming was unaffected (Argyropoulos, 2011).

### Imagery and visuospatial processing

The cerebellum is involved in pure imagery processes, both motor (Ryding et al., 1993; Naito et al., 2002) and visual (Ishai et al., 2000; Mellet et al., 2000). Indeed, patients affected by unilateral cerebellar stroke show slowed or impaired motor imagery (González et al., 2005; Battaglia et al., 2006). Moreover, cerebellar patients are impaired in tests of mental rotation of objects (a typical example of a visual imagery process) while, at the same time, failing to show significant deficits in tasks evaluating basic perceptual functioning or sensory discrimination (Molinari et al., 2004). Some cerebellar patients show purer perceptual alterations, such as hemispatial neglect (Silveri et al., 2001), and there is evidence that the cerebellum could be involved in metric judgment processes, as tested in the line bisection task (Fink et al., 2000).

The neural networks involved in imagery processes show a strong inter-individual and inter-trial variability; for example, Gerardin et al. (2000) found the cerebellum to be constantly activated during actual execution of motor actions, whilst there emerged strong inter-individual differences in its degree of activation during the execution of motor imagery tasks. Along the same lines, Grealy and Lee recently described a cerebellar patient found to be more impaired in monitoring imagined simple actions than in controlling the actual execution of the same actions (Grealy and Lee, 2011). Conversely, a different study reported cerebellar activation only during actual execution of motor acts and not while imaging the same acts (Nair et al., 2003) and a further one reported reduced cerebellar activity during imagined movements compared with actual execution of the same movements (Lotze et al., 1999). These heterogeneous results may be explained by individual differences, differences in the nature of the cerebellar lesions, and in the complexity or novelty of the tasks involved. However, another possible reason for the aforementioned differences could be that the cerebellum is actively engaged in *manipulating and monitoring* mental images rather than in *generating* them. In the last two studies (Lotze et al., 1999; Nair et al., 2003), the subjects were asked to imagine themselves executing relatively simple finger-tapping movements. Conversely, in the other study (Grealy and Lee, 2011) the patient was asked to imagine himself doing a pointing movement toward a specific location in space and to guess the amount of time required to execute the complete movement. Thus, in this case, the subject (who showed no difficulties of any kind in generating mental images) needed to actively monitor his motor imagery process and to estimate specific spatiotemporal information. Similarly, in the other reported studies linking the cerebellum with motor imagery, subjects were required to extrapolate some specific information from their imagery processes and/or to mentally imagine rather complex activities, such as playing tennis. In the same way, visual imagery tasks often require subjects to infer some kind of information from the mentally generated images and/or to actively manipulate these mental images (e.g., mental rotations). It is thus possible that the cerebellum is primarily engaged in manipulating mental images and in estimating spatiotemporal information related to dynamic motor imagery processes, whilst the pure generation of mental images probably does not rely primarily on cerebellar computations.

Furthermore, studies on hemicerebelloctomized rats, not displaying pure (declarative) spatial memory alteration, suggest that the cerebellum can play a major role in spatial navigation (Petrosini et al., 1998; Foti et al., 2010) and could be involved in developing procedural spatial search strategies.

### Decision-making and reasoning

The cerebellum is involved in decision-making under uncertainty (Blackwood et al., 2004) (probabilistic reasoning), which suggests that it can construct probabilistic models of external events. In a two-alternative forced-choice task condition, brain processing advances in four stages: processing of sensory information, option evaluation, intention formation, and, finally, action execution. In a recent MEG study (Guggisberg et al., 2007), the cerebellum and the inferior parietal cortex showed high frequency activity (gamma-band) during the intention formation and action execution stages (and, in some conditions, also during the option evaluation stage, mainly when all the options had the same value).

The cerebellum is also likely to be involved in reasoning processes of different types. For example, cerebellar activation has been observed during probabilistic and deductive reasoning (Osherson et al., 1998). Interestingly, deductive reasoning preferentially activates the left cerebellar hemisphere, while inductive reasoning activates the right cerebellar hemisphere (Goel and Dolan, 2004). Cerebellar activity in deductive reasoning seems to be independent of the presence/absence of semantic content (Goel et al., 2000), and also of its nature, concrete, or abstract (Goel and Dolan, 2001).

Although the meaning, if any, of cerebellar activation in reasoning is not fully understood, the cerebellum is thought to take part in creating and controlling adaptive working models of the environment, in cooperation with cortical structures, mainly the PFC (Vandervert, 2003; Vandervert et al., 2007). Indeed, there is interesting evidence that logical reasoning could be based on specific mental models and that, in turn, the internal structure of these models could directly influence the reasoning process (Johnson-Laird, 1980; Schaeken et al., 1996; Johnson-Laird, 2001). Therefore, the cerebellum could play an important role in manipulating the mental models required for logical reasoning.

## Mental processing: cerebellar involvement in neuropsychiatric disorders

Abnormal cerebellar processing can lead to alterations in mental functions. Cerebellar patients often show mood disorders, personality change, cognitive disorders, and dementia which may be integrated into the pathological frameworks of schizophrenia, depression, autism, and dyslexia. The rate of psychiatric morbidity associated with cerebellar degenerative diseases is about double that found in normal subjects (Leroi et al., 2002): 77% of patients with cerebellar degenerative diseases are affected by psychiatric disorders, compared with only 41% of neurologically healthy control subjects. Interestingly, the components of cognitive processing related to cerebellar activity also appear to be related to the pathogenesis of these diseases.

### Schizophrenia

Schizophrenia is a mental disorder characterized by a dissociation between internal representations and external reality. It is known that “cognitive dysmetria,” typical of psychoses like schizophrenia, is also observed in cerebellar patients. A role of the cerebellum in early onset schizophrenia was recently reported in a DTI study which revealed reduced fractional anisotropy in the white matter of the parietal association cortex and in the left cerebellar peduncle (Kyriakopoulos et al., 2008). Moreover, neurological soft signs in schizophrenic patients are inversely correlated with volume of the right cerebellar hemisphere (Bottmer et al., 2005). The cerebellar dysfunction may impair the ability of schizophrenic subjects to recognize an action on the basis of a subject's intention. Indeed, schizophrenic patients are not able to correctly estimate the sensory consequences of their own actions (Synofzik et al., 2010), a deficit usually observed in cerebellar patients. In other words, this means that the consequences of their actions are not in agreement with the expected sensory results of these actions and with the subject's intentions. This is hardly surprising given the predictive function of the cerebellum.

Neuroanatomically, there is evidence showing that damage to a CTCC could be the primary pathophysiological alteration in schizophrenic patients (Konarski et al., 2005). Several imaging studies (CT, MRI) have reported abnormal volume of the cerebellar vermis (either hypoplasia orhyperplasia), while others have reported global cerebellar atrophy. Cerebellar hypoactivation (or even non-activation) has been measured with fMRI in cognitive tasks involving the prefrontal-cerebellar loop, tasks such as the (1) Wisconsin Card-Sorting Test, (2) working memory [n–back] task, and (3) periodic sequence-learning tasks. An ontogenetic substrate can be traced back to abnormalities in infant motor development (IMD) and executive function development (Ridler et al., 2006). IMD and executive function development are normally associated with increased gray matter density (GMD) in the premotor cortex, striatum, and cerebellum, reinforcing the fronto-cerebellar network. Schizophrenic patients have delayed IMD and deficits in executive functions correlating with disruption of the fronto-cerebellar network. In postmortem studies, reduction in the size of the anterior vermis in schizophrenic patients is associated with reductions in the density and size of Purkinje cells. Moreover, the synaptogenesis process could be impaired, both for excitatory and inhibitory neurons, and a core alteration may concern the NMDA receptors and synaptic plasticity (Stephan et al., 2009).

Overall, in view of the reported impairment of the cerebellum and Purkinje cells, it can be hypothesized that the neural basis of schizophrenia might partially overlap that of autism (Boso et al., 2010). Considering the general function of the cerebellum, it is possible that schizophrenic patients are impaired in switching from an egocentric frame of reference to an allocentric one (Yakusheva et al., 2007). In agreement with this hypothesis, when asked to imagine an object from another perspective, schizophrenic individuals make more egocentric errors than do controls (Langdon et al., 2001; Shenton et al., 2001).

### Autism

Autism is a developmental disorder defined by three core features: (1) impairment in social interaction, (2) impairment in communication, with a delay in language acquisition, and (3) repetitive, stereotyped behaviors. More specifically, autistic subjects show a selective difficulty in understanding intentions and beliefs (Frith et al., 1991). Cerebellar patients and autistic subjects have shown a similar impairment in shifting attention between auditory and visual stimuli (Courchesne et al., 1994a). It is possible, given the critical role of the cerebellum in revealing differences between predictions elaborated by the cortex and the objective reality conveyed by experience, that dysfunction of CTCCs may prevent the detection of novelty and impair attention switching (Boso et al., 2010).

The cerebellum and the brainstem (including the inferior olive) are significantly smaller in autistic patients than in healthy controls (Hashimoto et al., 1995; Bauman and Kemper, 2005). The Purkinje cells of the cerebellum are reduced, primarily in the posterior inferior regions of the hemispheres. In the limbic system (hippocampus, amygdala, and enthorinal cortex), neurons are small and show increased cell-packing density.

Decreased exploration of the environment in autistic children (a typical autistic behavioral trait) is correlated with the magnitude of cerebellar hypoplasia of the vermal lobules VI–VII. The rate of stereotyped behavior is negatively correlated with the size of cerebellar vermal lobules VI–VII and positively correlated with frontal lobe volume in the same autistic subjects (Pierce and Courchesne, 2000). Interestingly, two types of cerebellar pathology have been identified in autism (Courchesne et al., 1994b): *hypoplasia* of the posterior vermal lobules VI and VII and *hyperplasia* of the same lobules. This is particularly relevant if we consider that vermal hyperplasia has also been found in subjects affected by Williams syndrome, a genetic disorder characterized by hyper sociability, social disinhibition, deficits in general intelligence, and visuospatial abilities, in the presence of preserved facial processing and language ability (Schmitt et al., 2001). Conversely, autism is characterized by social withdrawal and isolation. From the perspective of “social cognition,” these two pathologies can be seen as opposites (Riby and Hancock, 2008).

A neural response in the cerebellum, as in the visual cortex, is observable when processing a broad set of emotional facial expressions (happy, fearful, sad, angry, and disgusted faces) (Fusar-Poli et al., 2009). Conversely, the amygdala is selectively activated by happy, fearful, and sad faces, and the insula by disgusted and angry expressions. Alongside this evidence, an fMRI study has shown that the cerebellum is activated during implicit processing of facial expression, while temporal lobe regions are activated during explicit processing (Critchley et al., 2000a). Notably, when implicitly processing emotional expressions, subjects with high-functioning autistic disorders do not activate the left amygdala and the left cerebellum (Critchley et al., 2000b).

Abnormalities related to the autistic spectrum disorders have been found in spinocerebellar ataxia (SCA) patients (SCA3 and SCA6 patients), who show reduced performance on the Theory of Mind Test, in spite of showing normal attribution of social and emotional responses. These subjects also performed poorly in executive functions and memory tasks, but not in spatial and calculation tasks (Garrard et al., 2008). A previous study also found a specific cerebro-cerebellar network associated with “attribution of intention” tasks; this network is composed of the right medial and inferior parietal cortex, the temporal lobes, and the left cerebellum (Brunet et al., 2000).

### Depression

According to the DSM-IV (APA, 1994), a depressive disorder is characterized by a depressed mood and a loss of interest in daily activities. Depression can also be characterized by the presence of cognitive symptoms, such as weak working memory processing and impairment in executive functions (Fales et al., 2008).

A recent paper (Savitz and Drevets, 2009) reviewed neuroimaging studies (MRI and PET) in major depressive disorder (MDD) and bipolar disorder (BP), a mood disorder defined by the presence of manic episodes with (or without) depressive episodes. In MDD and BP, frequent findings are: (1) hypermetabolism with volume loss in the hippocampus and in the orbital and ventral PFC, and (2) hypometabolism in the dorsal PFC. Another study in MDD patients (Fitzgerald et al., 2008) reported constant hypoactivity in the cerebellum, insula, and frontal and temporal cortices. An increase in the activity of these areas correlates with anti-depressant treatment. Similarly, a recent work reported reduced regional homogeneity in the right insula and the left cerebellum in MMD patients and their siblings (Liu et al., 2010).

The cerebellum is likely to play an important role in modulating depressive symptoms; for example, it has been reported that high-frequency repetitive TMS over the cerebellum can cause a mood improvement in normal subjects and a significant reduction of depressive symptoms (Schutter and Honk, 2005). This is particularly interesting if we consider that this effect is probably dopamine-mediated; thus, the cerebellum is likely to exert a strong influence on the basal ganglia, regulating mood. Moreover, patients affected by SCA often show mild depression (Klinke et al., 2010), especially those with SCA1 and SCA6. A voxel-based morphometry study (Peng et al., 2010) reported GMD reductions in the bilateral insular cortex and left cerebellum in first-episode MDD patients, associated with decreased GMD also in the right medial and left lateral orbitofrontal cortex, right DLPFC, bilateral temporoparietal cortex, right superior temporal gyrus, and left parahyppocampal gyrus. Moreover, in MDD patients, the cerebellum, dorsal ACC, and precuneus show reduced connectivity with the orbital frontal cortex (Frodl et al., 2009).

As already explained, the cerebellum is connected with brain areas involved in emotional control, including the PFC and the hypothalamus (Zhu et al., 2006); however, a clear functional mechanism able to account for cerebellar involvement in mood regulation remains to be identified. Remarkably, MDD patients with psychotic disorders, when compared with MDD patients without psychotic disorders, show reduced perfusion of the left cerebellum and right superior frontal cortex, as well as increased perfusion of the left inferior PFC and caudate nucleus (Gonul et al., 2004). In addition to the intriguing aspects raised by this lateralization, these observations suggest that, in MDD, cerebellar activity correlates more closely with psychotic traits (e.g., delusions of control) than with “pure” depressive symptoms. The cerebellum is also involved in BP, with bipolar patients found to show poor emotional homeostasis, mania, and cognitive dysfunctions (Strakowski et al., 2005) and, on MRI scanning, a volume reduction in region V2 and V3 of the cerebellar vermis (Mills et al., 2005; Monkul et al., 2008).

### Dyslexia

Developmental dyslexia (DD) is characterized by a selective difficulty in acquiring reading skills, in spite of normal general intelligence (Habib, 2000). There are three main hypotheses regarding the core deficit responsible for DD symptoms: the magnosystem deficit hypothesis (MDH) (Stein, 2001), the phonological deficit hypothesis (PDH) (Ramus, 2003), and the cerebellar deficit hypothesis (CDH). The MDH takes its name from the “magnocellular neurons” of the lateral geniculate nucleus, which mostly feed the visual “dorsal stream” dedicated to the analysis of movement (the “where” pathway). Normal magnocellular function is necessary for high motion sensitivity and stable binocular perception which, in turn, are essential for proper development of orthographic skills. Many dyslexics show poor visual localization and their motion sensitivity is impaired, which suggests that abnormal development of the magnocellular system could play a pathogenetic role in DD. The PDH postulates that DD is caused by an impaired ability to represent and process speech sounds. The CDH, on the other hand, is based on the observation that dyslexic children show deficits in motor coordination, motor skills, and automatic processing, which suggests that a cerebellar dysfunction constitutes the neurological basis of the disease (Fawcett and Nicolson, 2004). Indeed, in a PET study, dyslexic subjects learning a motor sequence showed abnormalities in cerebellar activation during both automatic processing and new learning. An early cerebellar deficit has been hypothesized to impair the development of articulatory and writing skills (Nicolson et al., 2001b), and non-fluent articulation would engage more attentional resources, thereby impairing sensory feedback processing. It can thus be hypothesized that a cerebellar impairment causes a marked phonological deficit which, coupled with an automation deficit, results in DD. Cerebellar dysfunction can also explain the specific impairment in time estimation tasks shown by dyslexic subjects (Nicolson et al., 2001a). On the whole, the PDH can be seen as a part of the CDH. Conversely, the CDH does not completely explain the MDH, even though there is a subtype of DD characterized by magnocellular impairment (Tallal et al., 2006).

Dyslexics show small right cerebellar anterior lobes on MRI (Eckert et al., 2003), suggesting that a fronto-cerebellar circuit impairment could indeed cause the symptoms of DD. The right cerebellum is the brain area that best discriminates dyslexics from healthy control subjects (Pernet et al., 2009). Moreover, dyslexic subjects have symmetric cerebellar (as well as parietal and temporal cortex) gray matter, while healthy controls show a greater asymmetry (Hier et al., 1978; Rae et al., 2002). Therefore, there is substantial evidence that the cerebellum takes part in the pathogenesis of DD.

## Discussion

Recently, an impressive amount of new data has revealed a disconcerting heterogeneity of functional roles attributed to the cerebellum. Here we propose a unified framework that might provide a logical explanation of the numerous operations in which the cerebellum is involved.

### Different interpretations or disparate operational processes?

Ito, highlighting that the cerebellum can acquire forward models of a controlled object (e.g., the arm) through practice, proposes that the cerebellum uses internal models in order to adapt motor acts and mental activities to contextual information (Ito, 1993, 2008). By virtue of these models, the cerebellum is able to exert fast, precise, and automated control of well-learned movements. If we also think of thoughts and cognitive processes as controlled objects, the logical conclusion is that these same internal models can be applied to cognitive processing as well. This is hardly surprising, as mental products are virtual objects and there may not be much difference between a “thought” and a “thought to move.”

Along the same lines, Vandervert conjectures that the cerebellum is engaged in a dynamic interplay with working memory; his main idea is that repetitive working memory processes are actively adapted by the cerebellum and that this must result in better and faster attentional control and, consequently, in more precise and better timed cognitive processes (Vandervert, 2003, 2007; Vandervert et al., 2007).

Again, a comparable theoretical framework was advanced some years ago by Courchesne and Allen (1997). They hypothesized that the main function of the cerebellum is to predict the internal conditions required for different cognitive and motor activities and to rapidly and precisely set those conditions. Arguably, this “predict and prepare” function of the cerebellum must be implemented mainly unconsciously and automatically. Accordingly, the cerebellum is thought to be involved in implicit learning and, on the contrary, not to play a relevant role in declarative, explicit learning (Doyon et al., 1997). The cerebellum is likely to adopt a “trial-and-error” learning rule, unlike the cerebral cortex and basal ganglia, which seem to learn, respectively, through frequency-based and reward-based rules (Doya, 2000).

Finally, Ivry suggested that the cerebellum is involved in every task requiring precise timing processes (Ivry et al., 2002), including the production of skilled movements, eye-blink conditioning, duration discrimination of perceptual events, fast and precise regulation of attention and working memory, and some specific linguistic skills.

Other proposals are that the cerebellum is directly involved in cognitive/emotional processes, insofar as these are linked to some kind of motor or oculomotor activity (Doron et al., 2010), and that the main function of the cerebellum is to regulate the acquisition of sensory data across several sensory modalities, and thus to support various sensory, motor, and cognitive functions (Petacchi et al., 2005).

Clearly, although these frameworks are valid for interpreting data sets relative to specific experiments or methodologies, the link between these observations has remained largely speculative or unaddressed. Moreover, the involvement of the cerebellum in some cognitive functions, like attention, language, mental imagery, and reasoning, as well as in neuropsychiatric disorders and emotion, has remained obscure.

### A unified interpretation through the meta-levels hypothesis

What we suggest in this paper is that, as a result of modular connectivity with the cerebral cortex, causal relationships exist between the low- and high-level cognitive functions that the cerebellum is thought to play. Finally, we conjecture that failure of this system can lead to mental pathologies. The identified low-level functions, *timing, prediction, and learning*, directly implement structured cerebellar operations including forms of *working memory, error/novelty detection, and mental object manipulation*. On the one hand, timing, prediction, and learning have been related to cellular and network operations [e.g., see Medina and Mauk (2000); D'Angelo (2011); D'Angelo et al. (2011)], even though there is, as yet, no precise understanding of the mechanisms involved. On the other hand, these same functions may lie at the basis of more complex functions including *motor control, attention switching, language processing, imagery and visuospatial processing, decision-making, and reasoning*. Finally, major aspects of brain pathology can be predicted on the basis of these same low- and high-level functions. In some cases, direct evidence of these relationships has been demonstrated, while in others these inter-dependencies are still implicit, providing scope for testing of the hypothesis of cerebellar functioning based on its organization into meta-levels (Figure 4).

*Timing* seems the most fundamental of all the low-level functions. Timing may be directly explained on the basis of circuit mechanisms, whose cellular and synaptic components have been partly revealed [e.g., see D'Angelo and De Zeeuw (2009); D'Angelo et al. (2009)]. Timing is reflected in the ordering of complex sequences ordinarily processed by the cerebellum and it is deeply integrated with prediction and learning.

*Sensory prediction*, or, more generally, *prediction*, has been shown to predominate in motor control, attention switching, working memory, and language processing (Shadmehr and Mussa-Ivaldi, 2012), but it is also thought to intervene during imagery and visuospatial processing (which are also components of motor planning) and during decision-making and reasoning. So-called fluid intelligence, a form of cognitive control involved in executive functions (e.g., in the Tower of Hanoi test), rests on a dynamic sequence of selective attention, planning, storage in the working memory, hypothesis updating, attention redirecting, and so on. It is thought that the cerebellum is involved, at least when the problem is unusual and unexpected, according to its error/novelty-detection function. The circuits underlying prediction are unclear. It can be supposed that different inputs collide in proper time frames generating patterns that can, at some level, be recognized by pattern detectors. A model based on timing control in the granular layer and pattern detection in the molecular layer has been proposed (D'Angelo, 2011).

*Learning* in the cerebellum, and the original concepts related to this, probably need to be revised and extended in the light of new cellular and system physiology data. On the one hand, the cerebellar cortex and nuclei are clearly endowed with numerous mechanisms of long-term synaptic plasticity and therefore probably undergo changes during activity not just at the parallel fiber-Purkinje cell synapse, but also at other synaptic sites (Hansel et al., 2001; Jorntell and Hansel, 2006; Ohtsuki et al., 2009; Gao et al., 2012). These distributed changes could have very different meanings, for example that of controlling the spatiotemporal dynamics of signal processing in the granular layer and perceptron operations in Purkinje cells. On the other hand, functional imaging shows that the cerebellum is primarily involved when unknown problems or circumstances are encountered. This suggests that the cerebellum can incorporate specialized forms of procedural memory that can be reconfigured and transmitted to other brain areas. This learning can modify cerebellar spatiotemporal processing in terms of timing, pattern recognition, and coincidence detection, ultimately affecting the internal forward model and the consequent predictive properties. Cerebellar learning could contribute to working memory.

A puzzling implication of all this is that cerebellar processing might, ultimately, take part in generating *conscious and coherent representations of the world*, a function typically ascribed to the thalmo-cortical loops. Indeed, rapid continuous cerebellar processing in the cerebellar circuit through feedforward independent modules could enhance the immediate and continuous representation of events, which is one of the key aspects of consciousness (Addis et al., 2009; Nyberg et al., 2010; Szpunar, 2010, 2011). Also worth noting is the specific involvement of the cerebellum in elaborating *problems of a statistical nature*. In this case, it can be imagined that, by imposing an internal dynamic model, the cerebellum helps to automatically elaborate the trend in a complex data distribution on the basis of its previous acquisition of the most probable data sets. Interestingly, many biologically relevant problems have a statistical nature and the role of the cerebellum in this sense should be further explored.

A dramatic translation of low-level into high-level functions is observed in mental processing and even more clearly in the related dysfunction occurring in certain brain diseases. In *schizophrenia*, there is major failure of prediction-based comparison between internal and external representations, and of coordinate transformation and therefore manipulation of mental models. Similarly, *autism* involves a failure in redirecting attention, which can either be locked into internal contents or be hyperfocused on selective objects. In *depression*, psychotic symptoms may be regarded as a loss of internal coherence between internally and externally generated signals, with consequent dysregulation of mood homeostasis. Finally, in *dyslexia*, a combination of failures in the phonological loop involving working memory and recognition and manipulation of mental objects could be involved.

## Conclusions

The meta-levels hypothesis provides a key through which to order the multitude of manifestations of cerebellar physiology and pathology and reconcile the basic cerebellar functional mechanisms with the emerging properties of the network. The meta-levels hypothesis leads to testable predictions and opens the ways for new experimental designs. These can be broadly divided into those addressing (1) how the olivo-cerebellar system generates its internal representations and operations, (2) how the emerging functions derive from connections of the cerebellum with other brain structures, and (3) how dysfunction of the system could lead to pathology. In general, tools like genetic engineering in experimental animals, large-scale mathematical modeling and non-invasive stimulation/recording technology (like TMS and fMRI) in humans could provide valuable information. For example, genetic mutations impairing LTP (Long-Term Potentiation) or LTD (Long-Term Depression) could be used to investigate the potential role of the various forms of long-term synaptic plasticity expressed by cerebellar synapses not just with respect to motor learning but also with respect to cognitive processing extending through the CTCCs. Moreover, the role of cellular properties on circuit and system computations could be analyzed using detailed mathematical models, the CTCCs could be investigated by DTI, and their functional activation during specific tasks identified by fMRI. These same techniques could improve brain disease analysis and therapy. For example, cerebellar TMS has an impact on different pathologies, including Parkinson's disease (Koch et al., 2009a), epilepsy (Brighina et al., 2006), and stroke (Webster et al., 2006). Ultimately, this analysis could contribute to the development of a theory on global brain functioning in which the cerebellum is considered an integral part and not just a structure purely devoted to motor control.

### Conflict of interest statement

The authors declare that the research was conducted in the absence of any commercial or financial relationships that could be construed as a potential conflict of interest.

## Footnotes

^1^We need to clarify the terminology used. We here treat brain functions on a “neurological” level, where cognition and emotion are kept distinct. This differs from the definition used in neuroinformatics, in which “cognitive processing” refers to computational primitives—like pattern recognition, generalization, and abstraction by artificial neural networks. These definitions, furthermore, should not be confused with the tenet of classic cognitive psychology, which maintained that emotion is a category of cognition (Spitzer, 1998).

^2^A conceptual obstacle is that sensory-motor processing is thought to be mostly automatic (and unconscious) while cognition and emotion are bound to consciousness. Akin with this, the thalamo-cortical circuit is usually thought of as the site of conscious processing and the cerebro-cerebellar circuit as the site of automatic control. Indeed, the cerebellum probably does not take part in conscious representations directly, given that abnormalities of the cerebellum do not seem to impair consciousness. In their hypothesis, Tononi and Edelman suggest that the ability of circuits to generate consciousness lies in the strength of their internal connectivity, which is quite high in the cortex but comparatively much poorer in the cerebellum (Tononi and Edelman, 1998). This would explain why the cortex is capable of developing conscious percepts, while the cerebellum is not. However, the cerebellum is deeply interconnected in closed loops with the cerebral cortex over multiple independent lines involving associative as well as sensory-motor areas (see below), so that the activity of the two structures is strongly integrated.

^3^The cerebellum is made up of large compartments generally known as zones, which can be broken down into smaller compartments known as microzones. The demonstration, by microneurography, of cortical zones shows that each body part maps to specific points in the cerebellum, even though there are numerous repetitions of the basic map forming an arrangement that has been called “fractured somatotopy.” A different indication of compartmentalization is obtained by immune staining for certain types of protein (e.g., zebrin, NOS etc.). This reveals stripes oriented perpendicular to the cerebellar folds. Different markers generate different sets of stripes, and the widths and lengths vary as a function of location, but they all have the same general shape. Oscarsson in the 1970s proposed that these cortical zones can be partitioned into smaller units called microzones. In 2005, Apps and Garwicz showed that several microzones can be organized into a multizonal microcomplex. All microzones in the microcomplex are connected to the same group of deep cerebellar nuclei and inferior olivary neurons and correspond to the cerebellar modules defined by Voogd.

^4^According to a comparative anatomical, functional, and phylogenetic subdivision of the cerebellum, the vestibulocerebellum (archicerebellum) can be identified with the flocculo-nodular lobe, the spinocerebellum (paleocerebellum) with the rest of the vermis and para-vermal areas, and the cerebro-cerebellum (neocerebellum) with the cerebellar hemispheres. Hence, evolutionarily, there is a progressive increase in cerebro-cerebellar connections, which reach their maximum development in primates and humans, in parallel with the increased extension of the associative cortices.

^5^Motor controlis preprogrammed in a feed forward manner and is based on the coordination of elementary movements consisting of straight and curved segments. These “jerks” can be modulated in delay, duration, and strength. The analysis of a simple preprogrammed ballistic “jerk” movement, the saccadic movement needed to direct the eye toward a desired target, has shown that the cerebellum can indeed learn to control the beginning, the end, and the velocity of this elementary segment of movement.

^6^Cognitive control is the ability to direct mental processing toward complex targets. To achieve it, it is necessary to divert attention from actual sensory inputs, prioritize, and coordinate sequences of actions, and prolong this control until the target is achieved beyond immediate environmental interferences. Cognitive control allows regulation of executive functions, which can be automatic or controlled. Automatic executive functions are driven bottom-up through stimuli that activate internal behavioral modules, while the controlled executive functions operate in a top-down manner and imply choices based on general principles and experience. In general, once a controlled process is learned, it can be transformed into an automatic process, thereby accelerating its execution. The main properties of executive functions are that they are (1) goal-directed, (2) learned rather than innate, (3) multimodal; in addition, they (4) require generalization, (5) require working memory, (6) have limited capacity (only a few processes can be controlled at a time), and (7) require choices and selections to focus attention on a selected target. Executive control can associate, coordinate, and select multiple inputs and outputs, learning the procedure and generating behavioral flexibility and complexity. It should be noted that these aspects of cognitive control do not formally differ much from those of voluntary motor control.

^7^Emotional control, like cognitive control, has both a bottom-up and a top-down component. In the first case, a somato-visceral reaction (emotional response) is generated by detection of a significant pattern and the subject learns about it and subsequently elaborates an emotion (the James-Lange model). In the second case, the subject first elaborates an emotion and this then activates appropriate somato-visceral responses (the Cannon-Bard model).

## References

[B1] Ackermann H. Gräber S. Hertrich I. Daum I. (1997). Categorical speech perception in cerebellar disorders. Brain Lang. 60, 323–331 10.1006/brln.1997.1826 9344481

[B2] Addis D. R. Pan L. Vu M. A. Laiser N. Schacter D. L. (2009). Constructive episodic simulation of the future and the past: distinct subsystems of a core brain network mediate imagining and remembering. Neuropsychologia 47, 2222–2238 10.1016/j.neuropsychologia.2008.10.026 19041331

[B3] Ahissar E. Sosnik R. Haidarliu S. (2000). Transformation from temporal to rate coding in a somatosensory thalamocortical pathway. Nature 406, 302–306 10.1038/35018568 10917531

[B4] Alahyane N. Fonteille V. Urquizar C. Salemme R. Nighoghossian N. Pelisson D. (2008). Separate neural substrates in the human cerebellum for sensory-motor adaptation of reactive and of scanning voluntary saccades. Cerebellum 7, 595–601 10.1007/s12311-008-0065-5 19009327

[B5] Albus J. S. (1972). A theory of cerebellar function. Math. Biosci. 10, 25–61

[B6] Allen G. Buxton R. B. Wong E. C. Courchesne E. (1997). Attentional activation of the cerebellum independent of motor involvement. Science 275, 1940–1943 10.1126/science.275.5308.1940 9072973

[B7] Amodio D. M. Frith C. D. (2006). Meeting of minds: the medial frontal cortex and social cognition. Nature 7, 268–277 10.1038/nrn1884 16552413

[B8] Andersson G. Oscarsson O. (1978). Climbing fiber microzones in cerebellar vermis and their projection to different groups of cells in the lateral vestibular nucleus. Exp. Brain Res. 32, 565–579 689129 10.1007/BF00239553

[B9] Andreasen N. C. O'Leary D. S. Cizadlo T. Arndt S. Rezai K. Ponto L. L. (1996). Schizophrenia and cognitive dysmetria: a positron-emission tomography study of dysfunctional prefrontal-thalamic-cerebellar circuitry. Proc. Natl. Acad. Sci. U.S.A. 93, 9985–9990 8790444 10.1073/pnas.93.18.9985 PMC38542

[B10] Andreasen N. C. Paradiso S. O'Leary D. S. (1998). “Cognitive dysmetria” as an integrative theory of schizophrenia: a dysfunction in cortical-subcortical-cerebellar circuitry? Schizophr. Bull. 24, 203–218 9613621 10.1093/oxfordjournals.schbul.a033321

[B11] APA. (1994). Diagnostic and Statistical Manual of Mental Disorders 4. Washington, DC: American Psychiatric Association

[B12] Apps R. Garwicz M. (2005). Anatomical and physiological foundations of cerebellar information processing. Nat. Rev. Neurosci. 6, 297–311 10.1038/nrn1646 15803161

[B13] Apps R. Hawkes R. (2009). Cerebellar cortical organization: a one-map hypothesis. Nat. Rev. Neurosci. 10, 670–681 10.1038/nrn2698 19693030

[B14] Argyropoulos G. P. (2011). Cerebellar theta-burst stimulation selectively enhances lexical associative priming. Cerebellum 10, 540–550 10.1007/s12311-011-0269-y 21451999

[B15] Arriada-Mendicoa N. Otero-Siliceo E. Corona-Vazquez T. (1999). [Current concepts regarding the cerebellum and cognition]. Rev. Neurol. 29, 1075–1082 10637875

[B16] Baddeley A. Lewis V. Eldridge M. Thomson N. (1984). Attention and retrieval from long-term memory. J. Exp. Psychol. 113, 518–540

[B17] Balsters J. H. Ramnani N. (2008). Symbolic representations of action in the human cerebellum. Neuroimage 43, 388–398 10.1016/j.neuroimage.2008.07.010 18692577

[B18] Bartolo P. D. Gelfo F. Burello L. Giorgio A. D. Petrosini L. Granato A. (2011). Plastic changes in striatal fast-spiking interneurons following hemicerebellectomy and environmental enrichment. Cerebellum 10, 624–632 10.1007/s12311-011-0275-0 21509479

[B19] Battaglia F. Quartarone A. Ghilardi M. F. Dattola R. Bagnato S. Rizzo V. (2006). Unilateral cerebellar stroke disrupts movement preparation and motor imagery. Clin. Neurophysiol. 117, 1009–1016 10.1016/j.clinph.2006.01.008 16516543

[B20] Bauman M. L. Kemper T. L. (2005). Neuroanatomic observations of the brain in autism: a review and future directions. Int. J. Dev. Neurosci. 23, 183–187 10.1016/j.ijdevneu.2004.09.006 15749244

[B21] Bedny M. McGill M. Thompson-Schill L. S. (2008). Semantic adaptation and competition during word comprehension. Cereb. Cortex 18, 2574–2585 10.1093/cercor/bhn018 18308708 PMC2567420

[B22] Beldarrain M. G. García-Moncó J. Quintana J. Llorens V. Rodeño E. (1997). Diaschisis and neuropsychological performance after cerebellar stroke. Eur. Neurol. 37, 82–89 9058062 10.1159/000117415

[B23] Bell C. C. Han V. Sawtell N. B. (2008). Cerebellum-like structures and their implications for cerebellar function. Annu. Rev. Neurosci. 31, 1–24 10.1146/annurev.neuro.30.051606.094225 18275284

[B24] Blackwood N. Ffytche D. Simmons A. Bentallc R. Murray R. Howard R. (2004). The cerebellum and decision making under uncertainty. Brain Res. Cogn. Brain Res. 20, 46–53 10.1016/j.cogbrainres.2003.12.009 15130588

[B25] Blakemore S.-J. Frith C. D. Wolpert D. M. (2001). The cerebellum is involved in predicting the sensory consequences of action. Neuroreport 12, 1879–1884 11435916 10.1097/00001756-200107030-00023

[B26] Blakemore S.-J. Sirigu A. (2003). Action prediction in the cerebellum and in the parietal lobe. Exp. Brain Res. 153, 239–245 10.1007/s00221-003-1597-z 12955381

[B27] Blakemore S.-J. Wolpert D. M. Frith C. D. (1998a). Central cancellation of self-produced tickle sensation. Nature 1, 635–640 10.1038/2870 10196573

[B28] Blakemore S. J. Goodbody S. J. Wolpert D. M. (1998b). Predicting the consequences of our own actions: the role of sensorimotor context estimation. J. Neurosci. 18, 7511–7518 9736669 10.1523/JNEUROSCI.18-18-07511.1998 PMC6793221

[B29] Blakemore S.-J. Wolpert D. M. Frith C. D. (1999). The cerebellum contributes to somatosensory cortical activity during self-produced tactile stimulation. Neuroimage 10, 448–459 10.1006/nimg.1999.0478 10493902

[B30] Booth J. R. Wood L. Lu D. Houk J. C. Bitan T. (2007). The role of the basal ganglia and cerebellum in language processing. Brain Res. 1133, 136–144 10.1016/j.brainres.2006.11.074 17189619 PMC2424405

[B31] Boso M. Emanuele E. Prestori F. Politi P. Barale F. D'Angelo E. (2010). Autism and genius: is there a link? The involvement of central brain loops and hypotheses for functional testing. Funct. Neurol. 25, 15–20 20630121

[B32] Bostan A. C. Strick P. L. (2010). The cerebellum and basal ganglia are interconnected. Neuropsychol. Rev. 20, 261–270 10.1007/s11065-010-9143-9 20811947 PMC3325093

[B33] Bottmer C. Bachmann S. Pantel J. Essig M. Amann M. Schad L. R. (2005). Reduced cerebellar volume and neurological soft signs in first-episode schizophrenia. Psychiatry Res. 140, 239–250 10.1016/j.pscychresns.2005.02.011 16288852

[B34] Bower J. M. (1997). Is the cerebellum sensory for motor's sake, or motor for sensory's sake: the view from the whiskers of a rat? Prog. Brain Res. 114, 463–496 9193161 10.1016/s0079-6123(08)63381-6

[B35] Bower J. M. (2002). The organization of cerebellar cortical circuitry revisited: implications for function. Ann. N.Y. Acad. Sci. 978, 135–155 10.1111/j.1749-6632.2002.tb07562.x 12582048

[B36] Brighina F. Daniele O. Piazza A. Giglia G. Fierro B. (2006). Hemispheric cerebellar rTMS to treat drug-resistant epilepsy: case reports. Neurosci. Lett. 397, 229–233 10.1016/j.neulet.2005.12.050 16426754

[B37] Brunet E. Sarfati Y. Hardy-Bayle M.-C. Decety J. (2000). A PET investigation of the attribution of intentions with a nonverbal task. Neuroimage 11, 157–166 10.1006/nimg.1999.0525 10679187

[B38] Burguière E. Arleo A. Hojjati Mr. Elgersma Y. Zeeuw C. I. D. Berthoz A. (2005). Spatial navigation impairment in mice lacking cerebellar LTD: a motor adaptation deficit. Nature 8, 1292–1294 10.1038/nn1532 16136042

[B39] Buzsaki G. (2006). Rhythms of the Brain. New York, NY: Oxford University Press US

[B40] Cerminara N. L. (2010). Cerebellar modules: individual or composite entities? J. Neurosci. 30, 16065–16067 10.1523/JNEUROSCI.4823-10.2010 21123553 PMC6634852

[B41] Churchland P. (1998). Toward a Neurobiology of the Mind. London, England: mMIT Press

[B42] Churchland P. Sejinowski T. (1992). The Computational Brain. Cambridge, MA: MIT Press

[B43] Clower D. M. Dum R. P. Strick P. L. (2005). Basal ganglia and cerebellar inputs to “AIP”. Cereb. Cortex 15, 913–920 10.1093/cercor/bhh190 15459083

[B44] Clower D. M. West R. A. Lynch J. C. Strick P. L. (2001). The inferior parietal lobule is the target of output from the superior colliculus, hippocampus, and cerebellum. J. Neurosci. 21, 6283–6291 11487651 10.1523/JNEUROSCI.21-16-06283.2001 PMC6763148

[B45] Coghill R. C. Sang C. N. Maisog J. M. Iadarola M. J. (1999). Pain intensity processing within the human brain: a bilateral, distributed mechanism. J. Neurophysiol. 82, 1934–1943 10515983 10.1152/jn.1999.82.4.1934

[B46] Colnaghi S. Ramat S. D'Angelo E. Versino M. (2010). Transcranial magnetic stimulation over the cerebellum and eye movements: state of the art. Funct. Neurol. 25, 165–171 21232213

[B47] Connor L. T. Braby T. D. Snyder A. Z. Lewis C. Blasi V. Corbetta M. (2006). Cerebellar activity switches hemispheres with cerebral recovery in aphasia. Neuropsychologia 44, 171–177 10.1016/j.neuropsychologia.2005.05.019 16019040

[B48] Cotterill R. M. (2001). Cooperation of the basal ganglia, cerebellum, sensory cerebrum and hippocampus: possible implications for cognition, consciousness, intelligence and creativity. Prog. Neurobiol. 64, 1–33 10.1016/S0301-0082(00)00058-7 11250060

[B49] Courchesne E. Allen G. (1997). Prediction and preparation, fundamental functions of the cerebellum. Learn. Mem. 4, 1–35 10.1101/lm.4.1.1 10456051

[B50] Courchesne E. Townsend J. Akshoomoff N. A. Saitoh O. Yeung-Courchesne R. Lincoln A. J. (1994a). Impairment in shifting attention in autistic and cerebellar patients. Behav. Neurosci. 108, 848–865 7826509 10.1037//0735-7044.108.5.848

[B51] Courchesne E. Townsend J. Saitoh O. (1994b). The brain in infantile autism Posterior fossa structures are abnormal. Neurology 44, 214–223 8309561 10.1212/wnl.44.2.214

[B52] Craik F. I. Govoni R. Naveh-Benjamin M. Anderson N. D. (1996). The effects of divided attention on encoding and retrieval processes in human memory. J. Exp. Psychol. 125, 159–180 8683192 10.1037//0096-3445.125.2.159

[B53] Crespo-Facorro B. Paradiso S. Andreasen N. C. O'Leary D. S. Watkins G. L. Ponto L. L. B. (1999). Recalling word lists reveals “cognitive dysmetria” in schizophrenia: a positron emission tomography study. Am. J. Psychiatry 156, 386–392 10080553 10.1176/ajp.156.3.386

[B54] Critchley H. Daly E. Phillips M. Brammer M. Bullmore E. Williams S. (2000a). Explicit and implicit neural mechanisms for processing of social information from facial expressions: a functional magnetic resonance imaging study. Hum. Brain Mapp. 9, 93–105 10.1002/(SICI)1097-0193(200002)9:2<93::AID-HBM4>3.0.CO;2-Z 10680766 PMC6872127

[B55] Critchley H. D. Daly E. M. Bullmore E. T. Williams S. C. Amelsvoort T. V. Robertson D. M. (2000b). The functional neuroanatomy of social behaviour changes in cerebral blood flow when people with autistic disorder process facial expressions. Brain 123, 2203–2212 10.1093/brain/123.11.2203 11050021

[B56] Cui S.-Z. Li E.-Z. Zang Y.-F. Weng X.-C. Ivry R. Wang J.-J. (2000). Both sides of human cerebellum involved in preparation and execution of sequential movements. Neuroreport 11, 1–5 11117502 10.1097/00001756-200011270-00049

[B57] D'Angelo E. (2010a). Neuronal circuit function and dysfunction in the cerebellum: from neurons to integrated control. Funct. Neurol. 25, 125–127 21232207

[B58] D'Angelo E. (2010b). Rebuilding cerebellar network computations from cellular neurophysiology. Front. Cell. Neurosci. 4:131 10.3389/fncel.2010.00131 21103017 PMC2987656

[B59] D'Angelo E. (2011). Neural circuits of the cerebellum: hypothesis for function. J. Integr. Neurosci. 10, 317–352 21960306 10.1142/S0219635211002762

[B60] D'Angelo E. De Zeeuw C. I. (2009). Timing and plasticity in the cerebellum: focus on the granular layer. Trends Neurosci. 32, 30–40 10.1016/j.tins.2008.09.007 18977038

[B61] D'Angelo E. Koekkoek S. K. Lombardo P. Solinas S. Ros E. Garrido J. (2009). Timing in the cerebellum: oscillations and resonance in the granular layer. Neuroscience 162, 805–815 10.1016/j.neuroscience.2009.01.048 19409229

[B62] D'Angelo E. Mazzarello P. Prestori F. Mapelli J. Solinas S. Lombardo P. (2010). The cerebellar network: from structure to function and dynamics. Brain Res. Rev. 66, 5–15 10.1016/j.brainresrev.2010.10.002 20950649

[B63] D'Angelo E. Mazzarello P. Prestori F. Mapelli J. Solinas S. Lombardo P. (2011). The cerebellar network: from structure to function and dynamics. Brain Res. Rev. 66, 5–15 20950649 10.1016/j.brainresrev.2010.10.002

[B64] Dean P. Porrill J. Stone J. V. (2004). Visual awareness and the cerebellum: possible role of decorrelation control Prog. Brain Res. 144, 61–75 10.1016/S0079-6123(03)14404-4 14650840

[B65] Debas K. Carrier J. Orban P. Barakat M. Lungu O. Vandewalle G. (2010). Brain plasticity related to the consolidation of motor sequence learning and motor adaptation. Proc. Natl. Acad. Sci. U.S.A. 107, 17839–17844 10.1073/pnas.1013176107 20876115 PMC2955095

[B66] Desmond J. E. Chen S. H. Perry B. Shieh M. (2005). Cerebellar transcranial magnetic stimulation impairs verbal working memory. Ann. Neurol. 58, 553–560 10.1002/ana.20604 16178033

[B67] Desmond J. E. Gabrieli J. D. Wagner A. D. Ginier B. L. Glover G. H. (1997). Lobular patterns of cerebellar activation in verbal working-memory and finger-tapping tasks as revealed by functional, MRI. J. Neurosci. 17, 9675–9685 9391022 10.1523/JNEUROSCI.17-24-09675.1997 PMC6573411

[B68] De Zeeuw C. I. Hoebeek F. E. Bosman L. W. Schonewille M. Witter L. Koekkoek S. K. (2011). Spatiotemporal firing patterns in the cerebellum. Nat. Rev. Neurosci. 12, 327–344 10.1038/nrn3011 21544091

[B69] Diedrichsen J. Shadmehr R. Ivry R. B. (2010). The coordination of movement: optimal feedback control and beyond. Trends Cogn. Sci. 14, 31–39 10.1016/j.tics.2009.11.004 20005767 PMC4350769

[B70] Diener H.-C. Dichgans J. (1992). Pathophysiology of cerebellar ataxia. Mov. Disord. 7, 95–109 10.1002/mds.870070202 1584245

[B71] Dieudonné S. Dumoulin A. (2000). Serotonin-driven long-range inhibitory connections in the cerebellar cortex. J. Neurosci. 20, 1837–1848 10684885 10.1523/JNEUROSCI.20-05-01837.2000 PMC6772906

[B72] Dolan R. J. Bench C. J. Liddle P. F. Friston K. J. Frith C. D. Grasby P. M. (1993). Dorsolateral prefrontal cortex dysfunction in the major psychoses; symptom or disease specificity? J. Neurol. Neurosurg. Psychiatry 56, 1290–1294 8270929 10.1136/jnnp.56.12.1290 PMC1015376

[B73] Doron K. W. Funk C. M. Glickstein M. (2010). Fronto-cerebellar circuits and eye movement control: a diffusion imaging tractography study of human cortico-pontine projections. Brain Res. 1307, 63–71 10.1016/j.brainres.2009.10.029 19852951

[B74] Douglas R. J. Martin K. A. (2004). Neuronal circuits of the neocortex. Annu. Rev. Neurosci. 27, 419–451 10.1146/annurev.neuro.27.070203.144152 15217339

[B75] Doya K. (2000). Complementary roles of basal ganglia and cerebellum in learning and motor control. Curr. Opin. Neurobiol. 10, 732–739 10.1016/S0959-4388(00)00153-7 11240282

[B76] Doyon J. Gaudreau D. Laforce R. Jr. Castonguay M. Bédard P. J. Bédardd F. (1997). Role of the striatum, cerebellum, and frontal lobes in the learning of a visuomotor sequence. Brain Cogn. 34, 218–245 10.1006/brcg.1997.0899 9220087

[B77] Doyon J. Penhuneb V. Ungerleiderc L. G. (2003). Distinct contribution of the cortico-striatal and cortico-cerebellar systems to motor skill learning. Neuropsychologia 41, 252–262 10.1016/S0028-3932(02)00158-6 12457751

[B78] Doyon J. Song A. W. Karni A. Lalonde F. Adams M. M. Ungerleider L. G. (2002). Experience-dependent changes in cerebellar contributions to motor sequence learning. Proc. Natl. Acad. Sci. U.S.A. 99, 1017–1022 10.1073/pnas.022615199 11805340 PMC117423

[B79] Drepper J. Timmann D. Kolb F. P. Diener H. C. (1999). Expand+Non-motor associative learning in patients with isolated degenerative cerebellar disease. Brain 122, 87–97 10.1093/brain/122.1.87 10050897

[B80] Boisgueheneuc F. D. Levy R. Volle E. Seassau M. Duffau H. Kinkingnehun S. (2006). Functions of the left superior frontal gyrus in humans: a lesion study. Brain 129, 3315–3328 10.1093/brain/awl244 16984899

[B81] Dum R. P. Strick P. L. (2003). An unfolded map of the cerebellar dentate nucleus and its projections to the cerebral cortex. J. Neurophysiol. 89, 634–639 10.1152/jn.00626.2002 12522208

[B82] Eccles J. C. Ito M. Szentagothai J. (1967). The Cerebellum as a Neural Machine. Berlin, Heidelberg, New York: Springer-Verlag

[B83] Eckert M. A. Leonard C. M. Richards T. L. Aylward E. H. Thomson J. Berninger V. W. (2003). Anatomical correlates of dyslexia: frontal and cerebellar findings. Brain 126, 482–494 10.1093/brain/awg026 12538414

[B84] Elston G. N. DeFelipe J. (2002). Spine distribution in cortical pyramidal cells: a common organizational principle across species. Prog. Brain Res. 136, 109–133 12143375 10.1016/s0079-6123(02)36012-6

[B85] Fales C. L. Barch D. M. Rundle M. M. Mintun M. A. Snyder A. Z. Cohen J. D. (2008). Altered emotional interference processing in affective and cognitive-control brain circuitry in major depression. Biol. Psychiatry 63, 377–384 10.1016/j.biopsych.2007.06.012 17719567 PMC2268639

[B86] Fawcett A. Nicolson R. (2004). Dyslexia: the role of the cerebellum. Electron. J. Res. Edu. Psychol. 2, 35–58

[B87] Fink G. R. Marshall J. C. Shah N. J. Weiss P. H. Halligan P. W. Grosse-Ruyken M. (2000). Line bisection judgments implicate right parietal cortex and cerebellum as assessed by fMRI. Neurology 54, 1324–1331 10746605 10.1212/wnl.54.6.1324

[B88] Fitzgerald P. B. Laird A. R. Maller J. Daskalakis Z. J. (2008). A meta-analytic study of changes in brain activation in depression. Hum. Brain Mapp. 29, 683–695 10.1002/hbm.20426 17598168 PMC2873772

[B89] Flourens M.-J.-P. (1824). Recherches Expèrimentales sur les Propriétés et les Fonctions du Système Nerveux dans les Animaux Vertébres. Paris: Crevot

[B90] Foti F. Mandolesi L. Cutuli D. Laricchiuta D. Bartolo P. D. Gelfo F. (2010). Cerebellar damage loosens the strategic use of the spatial structure of the search space. Cerebellum 9, 29–41 10.1007/s12311-009-0134-4 19798544

[B91] Frings M. Maschke M. Gerwig M. Diener H.-C. Timmann D. (2006). Acquisition of simple auditory and visual sequences in cerebellar patients. Cerebellum 5, 206–211 10.1080/14734220600589251 16997752

[B92] Frith U. Morton J. Leslie A. M. (1991). The cognitive basis of a biological disorder: autism. Trends Neurosci. 14, 433–438 1722361 10.1016/0166-2236(91)90041-r

[B93] Frodl T. Bokde A. L. W. Scheuerecker J. Lisiecka D. Schoepf V. Hampel H. (2009). Functional connectivity bias of the orbitofrontal cortex in drug-free patients with major depression. Biol. Psychiatry 67, 161–167 10.1016/j.biopsych.2009.08.022 19811772

[B94] Fusar-Poli P. Placentino A. Carletti F. Landi P. Allen P. Surguladze S. (2009). Functional atlas of emotional faces processing: a voxel-based meta-analysis of 105 functional magnetic resonance imaging studies. J. Psychiatry Neurosci. 34, 418 19949718 PMC2783433

[B95] Gao Z. van Beugen B. J. De Zeeuw C. I. (2012). Distributed synergistic plasticity and cerebellar learning. Nat. Rev. Neurosci. 13, 619–635 10.1038/nrn3312 22895474

[B96] Garrard P. Martin N. H. Giunti P. Cipolotti L. (2008). Cognitive and social cognitive functioning in spinocerebellar ataxia. J. Neurol. 255, 398–405 10.1007/s00415-008-0680-6 18350360

[B97] Gebhart A. L. Petersen S. E. Thach W. T. (2002). Role of the posterolateral cerebellum in language. Ann. N.Y. Acad. Sci. 978, 318–333 10.1111/j.1749-6632.2002.tb07577.x 12582063

[B98] Gerardin E. Sirigu A. Lehéricy S. Poline J.-B. Gaymard B. Marsault C. (2000). Partially overlapping neural networks for real and imagined hand movements. Cereb. Cortex 10, 1093–1104 10.1093/cercor/10.11.1093 11053230

[B99] Ghajar J. Ivry R. B. (2009). The predictive brain state: asynchrony in disorders of attention? Neuroscientist 15, 232–243 10.1177/1073858408326429 19074688 PMC4342364

[B100] Goel V. Buchel C. Frith C. Dolan R. J. (2000). Dissociation of mechanisms underlying syllogistic reasoning. Neuroimage 12, 504–514 10.1006/nimg.2000.0636 11034858

[B101] Goel V. Dolan R. J. (2001). Functional neuroanatomy of three-term relational reasoning. Neuropsychologia 39, 901–909 10.1016/S0028-3932(01)00024-0 11516443

[B102] Goel V. Dolan R. J. (2004). Differential involvement of left prefrontal cortex in inductive and deductive reasoning. Cognition 93, 109–121 10.1016/j.cognition.2004.03.001 15178381

[B103] Goffart L. Chen L. Sparks D. (2003). Saccade dysmetria during functional perturbation of the caudal fastigial nucleus in the monkey. Annu. N.Y. Acad. Sci. 1004, 220–228 10.1196/annals.1303.019 14662461

[B104] Gonul A. S. Kula M. Bilgin A. G. Tutus A. Oguz A. (2004). The regional cerebral blood flow changes in major depressive disorder with and without psychotic features. Prog. Neuropsychopharmacol. Biol. Psychiatry 28, 1015–1021 10.1016/j.pnpbp.2004.05.036 15380862

[B105] González B. Rodríguez M. Ramirez C. Sabaté M. (2005). Disturbance of motor imagery after cerebellar stroke. Behav. Neurosci. 119, 622–626 10.1037/0735-7044.119.2.622 15839808

[B106] Gottwald B. Wilde B. Mihajlovic Z. Mehdorn H. M. (2004). Evidence for distinct cognitive deficits after focal cerebellarlesions. J. Neurol. Neurosurg. Psychiatry 75, 1524–1531 10.1136/jnnp.2003.018093 15489381 PMC1738803

[B107] Grealy M. A. Lee D. N. (2011). An automatic-voluntary dissociation and mental imagery disturbance following a cerebellar lesion. Neuropsychologia 49, 271–275 10.1016/j.neuropsychologia.2010.09.031 20932984

[B108] Grefkes C. Ritzla A. Zillesa K. Fink G. R. (2004). Human medial intraparietal cortex subserves visuomotor coordinate transformation. Neuroimage 23, 1494–1506 10.1016/j.neuroimage.2004.08.031 15589113

[B109] Grefkes C. Weiss P. H. Zilles K. Fink G. R. (2002). Crossmodal processing of object features in human anterior intraparietal cortex: an fMRI study implies equivalencies between humans and monkeys. Neuron 35, 173–184 10.1016/S0896-6273(02)00741-9 12123617

[B110] Guerrasio L. Quinet J. Büttner U. Goffart L. (2009). Fastigial oculomotor region and the control of foveation during fixation. J. Neurophysiol. 103, 1988–2001 10.1152/jn.00771.2009 20130038

[B111] Guggisberg A. G. Dalal S. S. Findlay A. M. Nagarajan S. S. (2007). High-frequency oscillations in distributed neural networks reveal the dynamics of human decision making. Front. Hum. Neurosci. 1:14 10.3389/neuro.09.014.2007 18958227 PMC2525986

[B112] Habas C. Kamdar N. Nguyen D. Keller K. Beckmann C. F. Menon V. (2009). Distinct cerebellar contributions to intrinsic connectivity networks. J. Neurosci. 29, 8586–8594 10.1523/JNEUROSCI.1868-09.2009 19571149 PMC2742620

[B113] Habib M. (2000). The neurological basis of developmental dyslexia. Brain 123, 2373–2399 10.1093/brain/123.12.2373 11099442

[B114] Haines D. D. E. May P. J. Dietrichs E. (1990). Neuronal connections between the cerebellar nuclei and hypothalamus in *Macaca fascicularis*: cerebello-visceral circuits. J. Comp. Neurol. 299, 106–122 10.1002/cne.902990108 1698835

[B115] Hansel C. Linden D. J. D'Angelo E. (2001). Beyond parallel fiber LTD: the diversity of synaptic and non-synaptic plasticity in the cerebellum. Nat. Neurosci. 4, 467–475 10.1038/87419 11319554

[B116] Hashimoto M. Ohtsuka K. (1995). Transcranial magnetic stimulation over the posterior cerebellum during visually guided saccades in man. Brain 118, 1185–1193 10.1093/brain/118.5.1185 7496779

[B117] Hashimoto T. Tayama M. Murakawa K. Yoshimoto T. Miyazaki M. Harada M. (1995). Development of the brainstem and cerebellum in autistic patients. J. Autism Dev. Disord. 25, 1–18 7608030 10.1007/BF02178163

[B118] He Y. Zang Y. (2004). Detecting functional connectivity of the cerebellum using Low Frequency Fluctuations (LFFs). Lect. Notes Comp. Sci. 3217, 907–915

[B119] Heath R. G. Harper J. W. (1974). Ascending projections of the cerebellar fastigial nucleus to the hippocampus, amygdala, and other temporal lobe sites: evoked potential and histological studies in monkeys and cats. Exp. Neurol. 45, 268–287 10.1016/0014-4886(74)90118-6 4422320

[B120] Hier D. B. LeMay M. Rosenberger P. B. Perlo V. P. (1978). Developmental dyslexia. Evidence for a subgroup with a reversal of cerebral asymmetry. Arch. Neurol. 35, 90–92 10.1001/archneur.1978.00500260028005 623535

[B121] Ishai A. Ungerleider L. G. Haxby J. V. (2000). Distributed neural systems for the generation of visual images. Neuron 28, 979–990 10.1016/S0896-6273(00)00168-9 11163281

[B122] Ito M. (1984). The Cerebellum and Neural Control. New York, NY: Raven Press

[B123] Ito M. (1993). Movement and thought: identical control mechanisms by the cerebellum. Trends Neurosci. 16, 448–450 discussion: 453–444. 7507615 10.1016/0166-2236(93)90073-u

[B124] Ito M. (2005). Bases and implications of learning in the cerebellum—adaptive control and internal model mechanism. Prog. Brain Res. 148, 95–109 10.1016/S0079-6123(04)48009-1 15661184

[B125] Ito M. (2008). Control of mental activities by internal models in the cerebellum. Nat. Rev. Neurosci. 9, 304–313 10.1038/nrn2332 18319727

[B126] Ivry R. B. (2000). Exploring the role of the cerebellum in sensory anticipation and timing: commentary on tesche and karhu. Hum. Brain Mapp. 9, 115–118 10.1002/(SICI)1097-0193(200003)9:3<115::AID-HBM1>3.0.CO;2-5 10739363 PMC6871905

[B127] Ivry R. B. Baldo J. V. (1992). Is the cerebellum involved in learning and cognition? Curr. Opin. Neurobiol. 2, 212–216 10.1016/0959-4388(92)90015-D 1638157

[B128] Ivry R. B. Keele S. W. (1989). Timing functions of the cerebellum. J. Cogn. Neurosci. 1, 136–152 10.1162/jocn.1989.1.2.136 23968462

[B129] Ivry R. B. Spencer R. M. Zelaznik H. N. Diedrichsen J. (2002). The cerebellum and event timing. Ann. N.Y. Acad. Sci. 978, 302–317 10.1111/j.1749-6632.2002.tb07576.x 12582062

[B130] Jackson P. L. Meltzoff A. N. Decety J. (2005). How do we perceive the pain of others? A window into the neural processes involved in empathy. Neuroimage 24, 771–779 10.1016/j.neuroimage.2004.09.006 15652312

[B131] Jacobson G. A. Rokni D. Yarom Y. (2008). A model of the olivo-cerebellar system as a temporal pattern generator. Trends Neurosci. 31, 617–625 10.1016/j.tins.2008.09.005 18952303

[B132] Johnson-Laird P. N. (1980). Mental models in cognitive science. Cogn. Sci. 4, 71–115

[B133] Johnson-Laird P. N. (2001). Mental models and deduction. Trends Cogn. Sci. 5, 434–442 10.1016/S1364-6613(00)01751-4 11707382

[B134] Jorntell H. Hansel C. (2006). Synaptic memories upside down: bidirectional plasticity at cerebellar parallel fiber-Purkinje cell synapses. Neuron 52, 227–238 10.1016/j.neuron.2006.09.032 17046686

[B135] Justus T. Ravizza S. M. Fiez J. A. Ivry R. B. (2005). Reduced phonological similarity effects in patients with damage to the cerebellum. Brain Lang. 95, 304–318 10.1016/j.bandl.2005.02.001 16246738 PMC2775087

[B136] Kelly R. M. Strick P. L. (2003). Cerebellar loops with motor cortex and prefrontal cortex of a nonhuman primate. J. Neurosci. 23, 8432–8444 12968006 10.1523/JNEUROSCI.23-23-08432.2003 PMC6740694

[B137] Kim C. H. Oh S. H. Lee J. H. Chang S. O. Kim J. Kim S. J. (2011). Lobule-specific membrane excitability of cerebellar Purkinje cells. J. Physiol. 590, 273–288 10.1113/jphysiol.2011.221846 22083600 PMC3285064

[B138] Klinke I. Minnerop M. Schmitz-Hübsch T. Hendriks M. Klockgether T. Wüllner U. (2010). Neuropsychological features of patients with Spinocerebellar Ataxia (SCA) types 1, 2, 3, and 6. Cerebellum 9, 433–442 10.1007/s12311-010-0183-8 20502998 PMC2949561

[B139] Koch G. Brusa L. Carrillo F. Gerfo E. L. Torriero S. Oliveri M. (2009a). Cerebellar magnetic stimulation decreases levodopa-induced dyskinesias in Parkinson disease. Neurology 73, 113–119 10.1212/WNL.0b013e3181ad5387 19597133

[B140] Koch G. Oliveri M. Caltagirone C. (2009b). Neural networks engaged in milliseconds and seconds time processing: evidence from transcranial magnetic stimulation and patients with cortical or subcortical dysfunction. Philos. Trans. R. Soc. Lond. B Biol. Sci. 364, 1907–1918 10.1098/rstb.2009.0018 19487193 PMC2685818

[B141] Konarski J. Z. McIntyre R. S. Grupp L. A. Kennedy S. H. (2005). Is the cerebellum relevant in the circuitry of neuropsychiatric disorders? J. Psychiatry Neurosci. 30, 178–186 15944742 PMC1089778

[B142] Krienen F. M. Buckner R. L. (2009). Segregated fronto-cerebellar circuits revealed by intrinsic functional connectivity. Cereb. Cortex 19, 2485–2497 10.1093/cercor/bhp135 19592571 PMC2742600

[B143] Kusunoki M. Gottlieb J. Goldberg M. E. (2000). The lateral intraparietal area as a salience map: the representation of abrupt onset, stimulus motion, and task relevance. Vis. Res. 40, 1459–1468 10.1016/S0042-6989(99)00212-6 10788652

[B144] Kyriakopoulos M. Vyasa N. S. Barkerb G. J. Chitnisb X. A. Frangoua S. (2008). A diffusion tensor imaging study of white matter in early-onset schizophrenia. Biol. Psychiatry 63, 519–523 10.1016/j.biopsych.2007.05.021 17662964

[B145] Lainé J. Axelrad H. (1998). Lugaro cells target basket and stellate cells in the cerebellar cortex. Neuroreport 9, 2399–2403 9694235 10.1097/00001756-199807130-00045

[B146] Langdon R. Coltheart M. Ward P. B. Catts S. V. (2001). Visual and cognitive perspective-taking impairments in schizophrenia: a failure of allocentric simulation? Cogn. Neuropsychiatry 6, 241–269

[B147] LeBeau F. E. El Manira A. Griller S. (2005). Tuning the network: modulation of neuronal microcircuits in the spinal cord and hippocampus. Trends Neurosci. 28, 552–561 10.1016/j.tins.2005.08.005 16112755

[B148] Lefèvre P. Quaia C. Opticana L. M. (1998). Distributed model of control of saccades by superior colliculus and cerebellum. Neural Netw. 11, 1175–1190 10.1016/S0893-6080(98)00071-9 12662742

[B149] Leggio M. G. Silveri M. C. Petrosini L. Molinari M. (2000). Phonological grouping is specifically affected in cerebellar patients: a verbal fluency study. J. Neurol. Neurosurg. Psychiatry 69, 102–106 10.1136/jnnp.69.1.102 10864613 PMC1736989

[B150] Leggio M. G. Tedesco A. M. Chiricozzi F. R. Clausi S. Orsini A. Molinari M. (2008). Cognitive sequencing impairment in patients with focal or atrophic cerebellar damage. Brain 131, 1332–1343 10.1093/brain/awn040 18334535

[B151] Leroi I. O'Hearn E. Marsh L. Lyketsos C. G. Rosenblatt A. Ross C. A. (2002). Psychopathology in patients with degenerative cerebellar diseases: a comparison to Huntington's disease. Am. J. Psichiatry 159, 1306–1314 10.1176/appi.ajp.159.8.1306 12153822

[B152] Liu Z. Xu C. Xu Y. Wang Y. Zhao B. Lv Y. (2010). Decreased regional homogeneity in insula and cerebellum: a resting-state fMRI study in patients with major depression and subjects at high risk for major depression. Psychiatry Res. 182, 211–215 10.1016/j.pscychresns.2010.03.004 20493670

[B153] Llinas R. Paré D. (1998). The Brain as a Closed System Modulated by the Senses. London, England: MIT Press

[B154] Llinás R. R. Roy S. (2009). The “prediction imperative” as the basis for self-awareness. Philos. Trans. R. Soc. Lond. B Biol. Sci. 364, 1301–1307 10.1098/rstb.2008.0309 19528011 PMC2666709

[B155] Lotze M. Montoya P. Erb M. Hülsmann E. Flor H. Klose U. (1999). Activation of cortical and cerebellar motor areas during executed and imagined hand movements: an fMRI study. J. Cogn. Neurosci. 11, 491–501 10511638 10.1162/089892999563553

[B156] Lubke J. Feldmeyer D. (2007). Excitatory signal flow and connectivity in a cortical column: focus on barrel cortex. Brain Struct. Funct. 212, 3–17 10.1007/s00429-007-0144-2 17717695

[B157] Luciani L. (1891). Il Cervelletto. Nuovi Studi di Fisiologia Normale e Patologica. Firenze: Le Monnier

[B158] Luft A. R. Manto M.-U. Taib N. O. B. (2005). Modulation of motor cortex excitability by sustained peripheral stimulation: the interaction between the motor cortex and the cerebellum. Cerebellum 4, 90–96 16035190 10.1080/14734220410019084

[B159] Maddox W. T. Aparicio P. Marchant N. L. Ivry R. B. (2005). Rule-based category learning is impaired in patients with Parkinson's disease but not in patients with cerebellar disorders. J. Cogn. Neurosci. 17, 707–723 10.1162/0898929053747630 15904539

[B160] Marien P. Saerens J. Nanhoe R. Moens E. Nagels G. Pickut B. A. (1996). Cerebellar induced aphasia: case report of cerebellar induced prefrontal aphasic language phenomena supported by SPECT findings. J. Neurol. Sci. 144, 34–43 8994102 10.1016/s0022-510x(96)00059-7

[B161] Marr D. (1969). A theory of cerebellar cortex. J. Physiol. 202, 437–470 5784296 10.1113/jphysiol.1969.sp008820 PMC1351491

[B163] Matell M. S. Meck W. H. (2004). Cortico-striatal circuits and interval timing: coincidence detection of oscillatory processes. Cogn. Brain Res. 21, 139–170 10.1016/j.cogbrainres.2004.06.012 15464348

[B164] Medina J. F. Mauk M. D. (2000). Computer simulation of cerebellar information processing. Nat. Neurosci. 3(Suppl.), 1205–1211 10.1038/81486 11127839

[B165] Mellet E. Tzourio-Mazoyer N. Bricogne S. Mazoyer B. Kosslyn S. M. Denis M. (2000). Functional anatomy of high-resolution visual mental imagery. J. Cogn. Neurosci. 12, 98–109 10769308 10.1162/08989290051137620

[B166] Miall R. C. Reckess G. Z. (2002). The cerebellum and the timing of coordinated eye and hand tracking. Brain Cogn. 48, 212–226 10.1006/brcg.2001.1314 11812043

[B167] Middleton F. A. Strick P. L. (2001). Cerebellar projections to the prefrontal cortex of the primate. J. Neurosci. 21, 700–712 11160449 10.1523/JNEUROSCI.21-02-00700.2001 PMC6763818

[B168] Milad M. R. Quirk G. J. (2002). Neurons in medial prefrontal cortex signal memory for fear extinction. Nature 420, 70–74 10.1038/nature01138 12422216

[B169] Mills N. P. DelBello M. P. Adler C. M. Strakowski S. M. (2005). MRI analysis of cerebellar vermal abnormalities in bipolar disorder. Am. J. Psichiatry 162, 1530–1532 10.1176/appi.ajp.162.8.1530 16055777

[B170] Moberget T. Karns C. M. Deouell L. Y. Lindgren M. Knight R. T. Ivry R. B. (2008). Detecting violations of sensory expectancies following cerebellar degeneration: a mismatch negativity study. Neuropsychologia 46, 2569–2579 10.1016/j.neuropsychologia.2008.03.016 18486157 PMC2588490

[B171] Molinari M. Filippini V. Leggio M. G. (2002). Neuronal plasticity of interrelated cerebellar and cortical networks. Neuroscience 3, 863–870 12031409 10.1016/s0306-4522(02)00024-6

[B172] Molinari M. Petrosini L. Misciagna S. Leggio M. G. (2004). Visuospatial abilities in cerebellar disorders. J. Neurol. Neurosurg. Psychiatry 75, 235–240 10.1136/jnnp.2003.015453 14742596 PMC1738892

[B173] Monkul E. S. Hatch J. P. Sassi R. B. Axelson D. Brambilla P. Nicoletti M. A. (2008). MRI study of the cerebellum in young bipolar patients. Prog. Neuropsychopharmacol. Biol. Psychiatry 32, 613–619 10.1016/j.pnpbp.2007.09.016 18272276 PMC2778760

[B174] Morgan M. A. Romanskib L. M. LeDoux J. E. (1993). Extinction of emotional learning: contribution of medial prefrontal cortex. Neurosci. Lett. 163, 109–111 10.1016/0304-3940(93)90241-C 8295722

[B175] Mugnaini E. Sekerková G. Martina M. (2011). The unipolar brush cell: a remarkable neuron finally receiving deserved attention. Brain Res. Rev. 66, 220–245 10.1016/j.brainresrev.2010.10.001 20937306 PMC3030675

[B176] Nair D. G. Purcott K. L. Fuchs A. Steinberg F. Kelso J. A. S. (2003). Cortical and cerebellar activity of the human brain during imagined and executed unimanual and bimanual action sequences: a functional MRI study. Brain Res. Cogn. Brain Res. 15, 250–260 10.1016/S0926-6410(02)00197-0 12527099

[B177] Naito E. Kochiyama T. Kitada R. Nakamura S. Matsumura M. Yonekura Y. (2002). Internally simulated movement sensations during motor imagery activate cortical motor areas and the cerebellum. J. Neurosci. 22, 3683–3691 11978844 10.1523/JNEUROSCI.22-09-03683.2002 PMC6758350

[B178] Nicolson R. I. Fawcett A. J. Dean P. (2001a). Developmental dyslexia: the cerebellar deficit hypothesis. Trends Neurosci. 24, 508–511 10.1016/S0166-2236(00)01896-8 11506881

[B179] Nicolson R. I. Fawcett A. J. Dean P. Zeffiro T. Eden G. (2001b). A TINS debate – Hindbrain versus the forebrain: a case for cerebellar deficit dyslexia: the cerebellar deficit hypothesis. Trends Neurosci. 24, 508–511 11506881 10.1016/s0166-2236(00)01896-8

[B180] Nyberg L. Kim A. S. Habib R. Levine B. Tulving E. (2010). Consciousness of subjective time in the brain. Proc. Natl. Acad. Sci. U.S.A. 107, 22356–22359 10.1073/pnas.1016823108 21135219 PMC3009795

[B181] Oberdick J. Sillitoe R. V. (2011). Cerebellar zones: history, development, and function. Cerebellum 10, 301–306 10.1007/s12311-011-0306-x 21822545

[B182] Ohtsuki G. Piochon C. Hansel C. (2009). Climbing fiber signaling and cerebellar gain control. Front. Cell. Neurosci. 3:4 10.3389/neuro.03.004.2009 19597563 PMC2708967

[B183] Oliveri M. Koch G. Torriero S. Caltagirone C. (2005). Increased facilitation of the primary motor cortex following 1 Hz repetitive transcranial magnetic stimulation of the contralateral cerebellum in normal humans. Neurosci. Lett. 376, 188–193 10.1016/j.neulet.2004.11.053 15721219

[B184] Osherson D. Perani D. Cappa S. Schnur T. Grassi F. Fazio F. (1998). Distinct brain loci in deductive versus probabilistic reasoning. Neuropsychologia 36, 369–376 10.1016/S0028-3932(97)00099-7 9665648

[B185] O'Reilly J. X. Mesulam M. M. Nobre A. C. (2008). The cerebellum predicts the timing of perceptual events. J. Neurosci. 28, 2252–2260 10.1523/JNEUROSCI.2742-07.2008 18305258 PMC6671847

[B186] Panouillères M. Neggers S. Gutteling T. Salemme R. Stigchel S. Geest J. (2011). Transcranial magnetic stimulation and motor plasticity in human lateral cerebellum: dual effect on saccadic adaptation. Hum. Brain Mapp. 33, 1512–1525 10.1002/hbm.21301 21692144 PMC6870392

[B187] Pascual-Leone A. Wassermann E. M. Grafman J. Hallett M. (1996). The role of the dorsolateral prefrontal cortex in implicit procedural learning. Exp. Brain Res. 107, 479–485 8821387 10.1007/BF00230427

[B188] Passot J.-B. Rondi-Reig L. Arleo A. (2009). Cerebellum and spatial cognition: a connectionist approach. Adv. Comput. Intell. Learn. 287–292

[B189] Pellionisz A. J. Llinàs R. (1982). Space-time representation in the brain the cerebellum as a predictive space-time metric tensor. Neuroscience 7, 2949–2970 7162624 10.1016/0306-4522(82)90224-x

[B190] Peng J. Liu J. Nie B. Li Y. Shan B. Wang G. (2010). Cerebral and cerebellar gray matter reduction in first-episode patients with major depressive disorder: a voxel-based morphometry study. Eur. J. Radiol. 80, 395–399 10.1016/j.ejrad.2010.04.006 20466498

[B191] Pernet C. R. Poline J. B. Demonet J. F. Rousselet G. A. (2009). Brain classification reveals the right cerebellum as the best biomarker of dyslexia. BMC Neurosci. 10:67 10.1186/1471-2202-10-67 19555471 PMC2713247

[B192] Petacchi A. Laird A. R. Fox P. T. Bower J. M. (2005). Cerebellum and auditory function: an ALE meta-analysis of functional neuroimaging studies. Hum. Brain Mapp. 25, 118–128 10.1002/hbm.20137 15846816 PMC6871682

[B193] Petrides M. (2000). The role of the mid-dorsolateral prefrontal cortex in working memory. Exp. Brain Res. 133, 44–54 10.1007/s002210000399 10933209

[B194] Petrosini L. (2007). “Do What I Do” and “Do How I Do”: different components of imitative learning are mediated by different neural structures. Neuroscientist 13, 335–348 10.1177/10738584070130040701 17644765

[B195] Petrosini L. Leggio M. G. Molinari M. (1998). The cerebellum in the spatial problem solving: a co-star or a guest star? Prog. Neurobiol. 56, 191–210 10.1016/S0301-0082(98)00036-7 9760701

[B196] Pierce K. Courchesne E. (2000). Evidence for a cerebellar role in reduced exploration and stereotyped behavior in autism. Biol. Psychiatry 49, 655–664 11313033 10.1016/s0006-3223(00)01008-8

[B197] Pijpers A. Apps R. Pardoe J. Voogd J. Ruigrok T. J. (2006). Precise spatial relationships between mossy fibers and climbing fibers in rat cerebellar cortical zones. J. Neurosci. 26, 12067–12080 10.1523/JNEUROSCI.2905-06.2006 17108180 PMC6674858

[B198] Ploghaus A. Becerra L. Borras C. Borsook D. (2003). Neural circuitry underlying pain modulation: expectation, hypnosis, placebo. Trends Cogn. Sci. 7, 197–200 10.1016/S1364-6613(03)00061-5 12757820

[B199] Pochon J.-B. Levy R. Poline J.-B. Crozier S. Lehéricy S. Pillon B. (2001). The role of dorsolateral prefrontal cortex in the preparation of forthcoming actions: an fMRI study. Cereb. Cortex 11, 260–266 10.1093/cercor/11.3.260 11230097

[B200] Prevosto V. Graf W. Ugolini G. (2010). Cerebellar inputs to intraparietal cortex areas LIP and MIP: functional frameworks for adaptive control of eye movements, reaching, and Arm/Eye/Head movement coordination. Cereb. Cortex 20, 214–228 10.1093/cercor/bhp091 19465740 PMC2860711

[B201] Rae C. Harasty J. A. Dzendrowskyj T. E. Talcott J. B. Simpson J. M. Blamire A. M. (2002). Cerebellar morphology in developmental dyslexia. Neuropsychologia 40, 1285–1292 10.1016/S0028-3932(01)00216-0 11931931

[B202] Ramnani N. Behrens T. E. Johansen-Berg H. Richter M. C. Pinsk M. A. Andersson J. L. (2006). The evolution of prefrontal inputs to the cortico-pontine system: diffusion imaging evidence from Macaque monkeys and humans. Cereb. Cortex 16, 811–818 10.1093/cercor/bhj024 16120793

[B203] Ramnani N. Owen A. M. (2004). Anterior prefrontal cortex: insights into function from anathomy and neuroimaging. Nature 5, 184–194 10.1038/nrn1343 14976518

[B204] Ramus F. (2003). Developmental dyslexia: specific phonological deficit or general sensorimotor dysfunction? Curr. Opin. Neurobiol. 13, 212–218 10.1016/S0959-4388(03)00035-7 12744976

[B205] Ravizza S. M. McCormick C. A. Schlerf J. E. Justus T. Ivry R. B. Fiez J. A. (2006). Cerebellar damage produces selective deficits in verbal working memory. Brain 129, 306–320 10.1093/brain/awh685 16317024

[B206] Riby D. M. Hancock P. J. B. (2008). Viewing it differently: social scene perception in Williams syndrome and Autism. Neuropsychologia 46, 2855–2860 10.1016/j.neuropsychologia.2008.05.003 18561959

[B207] Richter S. Kaiser O. Hein-Kropp C. Dimitrova A. Gizewski E. Beckc A. (2004). Preserved verb generation in patients with cerebellar atrophy. Neuropsychologia 42, 1235–1246 10.1016/j.neuropsychologia.2004.01.006 15178175

[B208] Ridderinkhof K. R. Ullsperger M. Crone E. A. Nieuwenhuis S. (2004). The role of the medial frontal cortex in cognitive control. Science 306, 443–447 10.1126/science.1100301 15486290

[B209] Ridler K. Veijola J. M. Tanskanen P. Miettunen J. Chitnis X. Suckling J. (2006). Fronto-cerebellar systems are associated with infant motor and adult executive functions in healthy adults but not in schizophrenia. Proc. Natl. Acad. Sci. U.S.A. 103, 15651–15656 10.1073/pnas.0602639103 17028177 PMC1636802

[B210] Rissman J. Eliassen J. C. Blumstein S. E. (2003). An event-related fMRI investigation of implicit semantic priming. J. Cogn. Neurosci. 15, 1160–1175 10.1162/089892903322598120 14709234

[B211] Robinson F. R. Straube A. Fuchs A. F. (1993). Role of the caudal fastigial nucleus in saccade generation. II. Effects of muscimol inactivation. J. Neurophysiol. 70, 1741–1758 8294950 10.1152/jn.1993.70.5.1741

[B212] Rouiller E. M. Liang F. Babalian A. Moret V. Wiesendanger M. (1994). Cerebellothalamocortical and pallidothalamocortical projections to the primary and supplementary motor cortical areas: a multiple tracing study in macaque monkeys. J. Comp. Neurol. 345, 185–213 10.1002/cne.903450204 7523459

[B213] Ruigrok T. J. H. (2010). Ins and outs of cerebellar modules. Cerebellum 10, 464–474 10.1007/s12311-010-0164-y 20232190 PMC3169761

[B214] Rushworth M. F. S. Behrens T. E. J. Johansen-Berg H. (2006). Connection patterns distinguish 3 regions of human parietal cortex. Cereb. Cortex 16, 1418–1430 10.1093/cercor/bhj079 16306320

[B215] Ryding E. Decety J. Sjöholm H. Stenberg G. Ingvar D. H. (1993). Motor imagery activates the cerebellum regionally. A SPECT rCBF study with 99mTc-HMPAO. Brain Res. Cogn. Brain Res. 1, 94–99 10.1016/0926-6410(93)90015-W 8513244

[B216] Sacchetti B. Scelfo B. Strata P. (2005). The cerebellum: synaptic changes and fear conditioning. Neuroscientist 11, 217–227 10.1177/1073858405276428 15911871

[B217] Salmi J. Pallesen K. J. Neuvonen T. Brattico E. Korvenoja A. Salonen O. (2010). Cognitive and motor loops of the human cerebro-cerebellar system. J. Cogn. Neurosci. 22, 2663–2676 10.1162/jocn.2009.21382 19925191

[B218] Savitz J. Drevets W. C. (2009). Bipolar and major depressive disorder: neuroimaging the developmental-degenerative divide. Neurosci. Biobehav. Rev. 33, 699–771 10.1016/j.neubiorev.2009.01.004 19428491 PMC2858318

[B219] Schaeken W. Johnson-Laird P. N. D'Ydewalle G. (1996). Mental models and temporal reasoning. Cognition 60, 205–234 10.1016/0010-0277(96)00708-1 8870513

[B220] Schmahmann J. D. (2004). Disorders of the cerebellum: ataxia, dysmetria of thought, and the cerebellar cognitive sffective syndrome. J. Neuropsychiatry Clin. Neurosci. 16, 367–378 10.1176/appi.neuropsych.16.3.367 15377747

[B221] Schmahmann J. D. Caplan D. (2006). Cognition, emotion and the cerebellum. Brain 129, 288–292 16434422 10.1093/brain/awh729

[B222] Schmahmann J. D. Sherman J. C. (1998). The cerebellar cognitive affective syndrome. Brain 121, 561–579 9577385 10.1093/brain/121.4.561

[B223] Schmitt J. E. Eliez S. Warsofsky I. S. Bellugi U. Reissa A. L. (2001). Enlarged cerebellar vermis in Williams syndrome. J. Psychiatr. Res. 35, 225–229 11578640 10.1016/s0022-3956(01)00024-3

[B224] Schutter D. J. Honk J. (2005). A framework for targeting alternative brain regions with repetitive transcranial magnetic stimulation in the treatment of depression. J. Psychiatry Neurosci. 30, 91–97 15798784 PMC551160

[B225] Schweizer T. A. Levine B. Rewilak D. O'Connor C. Turner G. Alexander M. P. (2007). Rehabilitation of executive functioning after focal damage to the cerebellum. Neurorehabil. Neural Repair 22, 72–77 10.1177/1545968307305303 17664355

[B226] Seger C. A. Desmond J. E. Glover G. H. Gabrieli J. D. E. (2000). Functional magnetic resonance imaging evidence for right-hemisphere involvement in processing unusual semantic relationships. Neuropsychology 14, 361–369 10928739 10.1037//0894-4105.14.3.361

[B227] Shadmehr R. Mussa-Ivaldi S. (2012). Biological Learning and Control. Cambridge: MIT Press

[B228] Shenton M. E. Dickey C. C. Frumin M. McCarley R. W. (2001). A review of MRI findings in schizophrenia. Schizophr. Res. 49, 1–52 10.1016/S0920-9964(01)00163-3 11343862 PMC2812015

[B229] Shin J. C. Ivry R. B. (2003). Spatial and temporal sequence learning in patients with Parkinson's disease or cerebellar lesions. J. Cogn. Neurosci. 15, 1232–1243 10.1162/089892903322598175 14709239

[B230] Silveri M. C. Misciagna S. Terrezza G. (2001). Right side neglect in right cerebellar lesion. J. Neurol. Neurosurg. Psychiatry 71, 114–117 10.1136/jnnp.71.1.114 11413276 PMC1737471

[B231] Smet H. J. D. Baillieux H. Deyn P. P. D. Mariën P. Paquier P. (2007). The cerebellum and language: the story so far. Folia Phoniatr. Logop. 59, 165–170 10.1159/000102927 17627124

[B232] Snider R. S. Maiti A. (1976). Cerebellar contributions to the papez circuit. J. Neurosci. Res. 2, 133–146 10.1002/jnr.490020204 950678

[B232a] Spitzer M. (1998). The Mind Within the Net: Model of Learning, Thinking and Acting. Cambridge: MIT Press

[B233] Stein J. (2001). The magnocellular theory of developmental dyslexia. Dyslexia 7, 12–36 10.1002/dys.186 11305228

[B234] Stephan K. E. Friston K. J. Frith C. D. (2009). Dysconnection in schizophrenia: from abnormal synaptic plasticity to failures of self-monitoring. Schizophr. Bull. 35, 509–527 10.1093/schbul/sbn176 19155345 PMC2669579

[B235] Strakowski S. M. Adler C. M. Holland S. K. Mills N. P. DelBello M. P. Eliassen J. C. (2005). Abnormal fMRI brain activation in euthymic bipolar disorder patients during a counting stroop interference task. Am. J. Psichiatry 162, 1697–1705 10.1176/appi.ajp.162.9.1697 16135630

[B236] Straube A. Buttner U. (2007). Neuro-Ophthalmology. Neuronal Control of Eye Movements. Magdeburg: Karger

[B237] Strick P. L. Dum R. P. Fiez J. A. (2009). Cerebellum and nonmotor function. Annu. Rev. Neurosci. 32, 413–434 10.1146/annurev.neuro.31.060407.125606 19555291

[B238] Synofzik M. Lindner A. Thier P. (2008). The cerebellum updates predictions about the visual consequences of one's behavior. Curr. Biol. 18, 814–818 10.1016/j.cub.2008.04.071 18514520

[B239] Synofzik M. Thier P. Leube D. T. Schlotterbeck P. Lindner A. (2010). Misattributions of agency in schizophrenia are based on imprecise predictions about the sensory consequences of one's actions. Brain 133, 262–271 10.1093/brain/awp291 19995870

[B240] Szpunar K. K. (2010). Evidence for an implicit influence of memory on future thinking. Mem. Cognit. 38, 531–540 10.3758/MC.38.5.531 20551334

[B241] Szpunar K. K. (2011). On subjective time. Cortex 47, 409–411 10.1016/j.cortex.2010.07.008 20801435

[B242] Szwed M. Bagdasarian K. Ahissar E. (2003). Encoding of vibrissal active touch. Neuron 40, 621–630 10.1016/S0896-6273(03)00671-8 14642284

[B243] Takagi M. Zee D. S. Tamargo R. J. (1999). Effects of lesions of the oculomotor cerebellar vermis on eye movements in primate: smooth pursuit. J. Neurophysiol. 83, 2047–2062 10758115 10.1152/jn.2000.83.4.2047

[B244] Tallal P. Miller S. Holly F. R. (2006). Neurobiological basis of speech: a case for the preeminence of temporal processing. Ann. N.Y. Acad. Sci. 682, 27–47 7686725 10.1111/j.1749-6632.1993.tb22957.x

[B245] Tavano A. Borgatti R. (2010). Evidence for a link among cognition, language and emotion in cerebellar malformations. Cortex 46, 907–918 10.1016/j.cortex.2009.07.017 19857864

[B246] Tilikete C. Koene A. Nighoghossian N. Vighetto A. Pélisson D. (2006). Saccadic lateropulsion in Wallenberg syndrome: a window to access cerebellar control of saccades? Exp. Brain Res. 174, 555–565 10.1007/s00221-006-0495-6 16680426

[B247] Tononi G. Edelman G. M. (1998). Consciousness and complexity. Science 282, 1846–1851 10.1126/science.282.5395.1846 9836628

[B248] Townsend J. Courchesne E. Covington J. Westerfield M. Harris N. S. Lyden P. (1999). Spatial attention deficits in patients with acquired or developmental cerebellar abnormality. J. Neurosci. 19, 5632–5643 10377369 10.1523/JNEUROSCI.19-13-05632.1999 PMC6782343

[B249] Tunik E. Frey S. H. Grafton S. T. (2005). Virtual lesions of the anterior intraparietal area disrupt goal-dependent on-line adjustments of grasp. Nature 8, 505–511 10.1038/nn1430 15778711 PMC10719865

[B250] Turner B. M. Paradiso S. Marvel C. L. Pierson R. BolesPonto L. L. Hichwa R. D. (2007). The cerebellum and emotional experience. Neuropsychologia 45, 1331–1341 10.1016/j.neuropsychologia.2006.09.023 17123557 PMC1868674

[B251] Uusisaari M. Y. Knopfel T. (2012). Diversity of neuronal elements and circuitry in the cerebellar nuclei. Cerebellum 11, 420–421 10.1007/s12311-011-0350-6 22278661

[B252] Vandervert L. R. (2003). How working memory and cognitive modeling functions of the cerebellum contribute to discoveries in mathematics. New Ideas Psychol. 21, 15–29

[B253] Vandervert L. R. (2007). Cognitive functions of the cerebellum explain how Ericsson's deliberate practice produces giftedness. High Abil. Stud. 18, 89–92

[B254] Vandervert L. R. Schimpf P. H. Liu H. (2007). How working memory and the cerebellum collaborate to produce creativity and innovation. Creat. Res. J. 19, 1–18

[B255] Voogd J. (2010). Cerebellar zones: a personal history. Cerebellum 10, 334–350 20967577 10.1007/s12311-010-0221-6 PMC3169774

[B256] Wang S. Wu D.-C. Ding M.-P. Li Q. Zhuge Z.-B. Zhang S.-H. (2008). Low-frequency stimulation of cerebellar fastigial nucleus inhibits amygdaloid kindling acquisition in Sprague–Dawley rats. Neurobiol. Dis. 29, 52–58 10.1016/j.nbd.2007.07.027 17904855

[B257] Watson T. C. Jones M. W. Apps R. (2009). Electrophysiological mapping of novel prefrontal–cerebellar pathways. Front. Integr. Neurosci. 3:18 10.3389/neuro.07.018.2009 19738932 PMC2737490

[B258] Webster B. R. Celnik P. A. Cohen L. G. (2006). Noninvasive brain stimulation in stroke rehabilitation. NeuroRX 3, 474–481 10.1016/j.nurx.2006.07.008 17012061 PMC3593409

[B259] Weinberger D. R. Berman K. F. Illowsky B. P. (1988). Physiological dysfunction of dorsolateral prefrontal cortex in schizophrenia, I. I. I. A New Cohort and Evidence for a Monoaminergic Mechanism. Arch. Gen. Psychiatry 45, 609–615 10.1001/archpsyc.1988.01800310013001 3382320

[B260] Weinberger D. R. Berman K. F. Zec R. F. (1986). Physiologic dysfunction of dorsolateral prefrontal cortex in schizophrenia, I. Regional cerebral blood flow evidence. Arch. Gen. Psychiatry 43, 114–124 10.1001/archpsyc.1986.01800020020004 3947207

[B261] Wiech K. Ploner M. Tracey I. (2008). Neurocognitive aspects of pain perception. Trends Cogn. Sci. 12, 306–313 10.1016/j.tics.2008.05.005 18606561

[B262] Wolpert D. M. Miall R. C. Kawato M. (1998). Internal models in the cerebellum. Trends Cogn. Sci. 2, 338–347 21227230 10.1016/s1364-6613(98)01221-2

[B263] Xiang H. Lin C. Ma X. Zhang Z. Bower J. M. Weng X. (2003). Involvement of the cerebellum in semantic discrimination: an fMRI study. Hum. Brain Mapp. 18, 208–214 10.1002/hbm.10095 12599279 PMC6872119

[B264] Xivry J.-J. Lefèvre P. (2007). Saccades and pursuit: two outcomes of a single sensorimotor process. J. Physiol. 584, 11–23 10.1113/jphysiol.2007.139881 17690138 PMC2277072

[B265] Yakusheva T. A. Shaikh A. G. Green A. M. Blazquez P. M. Dickman J. D. Angelaki D. E. (2007). Purkinje cells in posterior cerebellar vermis encode motion in an inertial reference frame. Neuron 54, 973–985 10.1016/j.neuron.2007.06.003 17582336

[B266] Zhu J.-N. Yung W.-H. Chow B. K.-C. Chand Y.-S. Wanga J.-J. (2006). The cerebellar-hypothalamic circuits: Potential pathways underlying cerebellar involvement in somatic-visceral integration. Brain Res. Rev. 52, 93–106 10.1016/j.brainresrev.2006.01.003 16497381

